# Whole-Genome Sequencing in Diagnostics of Unsolved Cases of Inherited Retinal Diseases

**DOI:** 10.3390/ijms27209022

**Published:** 2026-10-12

**Authors:** Natalya Ogorodova, Anna Stepanova, Polina Geletskaia, Vitaly Kadyshev, Rena Zinchenko, Svetlana Kuznetsova, Olga Khalanskaya, Alexandra Monakhova, Nikita Beskorovainy, Victoria Zabnenkova, Oksana Ryzhkova, Konstantin Severinov, Igor Bychkov, Alexander Polyakov, Sergey Kutsev, Olga Shchagina

**Affiliations:** 1 Research Centre for Medical Genetics, 115522 Moscow, Russia; 2 Biotech Campus Limited Liability Company, 117437 Moscow, Russia

**Keywords:** whole-genome sequencing, inherited retinal diseases, deep intronic variants, structural rearrangements, copy number variants

## Abstract

The study purpose was to assess the diagnostic value of whole-genome sequencing (WGS) in identifying candidate genetic variants in patients with inherited retinal diseases (IRDs). We investigated 107 unrelated Russian patients with retinal diseases who carried single pathogenic variants in any of nine common autosomal recessive IRD genes *(ABCA4, USH2A, EYS, CRB1, RPE65, CNGB3, CNGA3, CEP290,* and *GUCY2D*). WGS was performed for all patients; candidate splice variants were functionally validated using minigene assays. The total diagnostic yield was 40.2% (43/107). In 25.2% of cases (27/107), the diagnosis was established by identifying a second variant in one of the following candidate genes: *EYS* (*n* = 9), *USH2A* (*n* = 8), *ABCA4* (*n* = 5), *CNGB3* (*n* = 3), *CRB1* and *CEP290* (*n* = 1 each). In 15.0% of cases (16/107), the diagnosis was reclassified by variants in alternative IRD genes (autosomal dominant, X-linked, mitochondrial, or syndromic). The majority of second variants detected in AR cases were CNVs (55.5%, 15 patients), followed by non-coding variants (26.0%, seven), structural rearrangements (14.8%, four) and exonic point variant (3.7%, one). Two recurrent CNVs were observed: a gross deletion of exons 22–24 in *USH2A* (*n* = 4) and a deletion partially affecting exon 19 of *EYS* (*n* = 4). Structural variants were mostly represented by delINV rearrangements (*n* = 5). Minigene assays confirmed that three novel deep intronic variants (in *CHM*, *EYS*, and *CEP290*) and one non-canonical splice-acceptor variant in *USH2A* which disrupt splicing, causes pseudoexon inclusion with premature termination or in-frame exon skipping respectively. Thus, WGS improves the diagnostic yield in IRDs by detecting variants missed by targeted sequencing, supporting its clinical utility in ophthalmologic practice.

## 1. Introduction

Inherited retinal diseases (IRDs) are a clinically and genetically heterogeneous group of disorders characterized by degenerative changes in the retina, involving the photoreceptors or the retinal pigment epithelium. The average global prevalence of IRDs is estimated at approximately one in 3500 individuals [1]. Autosomal recessive forms account for 50% to 85% of all molecularly confirmed diagnoses [2,3].

To date, more than 300 genes have been described as causative for various forms of IRDs (https://sph.uth.edu/Retnet/ (accessed on 3 May 2026)). A significant proportion of IRDs are caused by the pathology of cilia and centrosomes, which in turn leads to systemic manifestations, i.e., syndromic forms (ciliopathies). In addition, an important component of the pathogenesis of IRDs is impairment of the enzyme systems involved in the visual cycle and phototransduction cascade [4]. Given this extensive genetic heterogeneity, high-throughput sequencing has become the most common approach for molecular genetic diagnostics.

Gene panel sequencing is widely used due to its cost-effectiveness, as well as the relative simplicity of data interpretation and storage. The diagnostic yield of panel sequencing typically ranges from 40% to 76% [5]. The application of another targeted enrichment method, whole-exome sequencing (WES), enables the identification of novel candidate genes that may be associated with IRDs. However, the diagnostic effectivity of WES is generally comparable to that of custom gene panels [6,7].

After targeted sequencing, in approximately 5–10% of patients, a single pathogenic or likely pathogenic variant in one or more genes associated with autosomal recessive retinal disease is typically detected [8,9,10]. The interpretation of such cases remains challenging, as it is difficult to determine whether the detected variant is causative or represents incidental carrier status, with the underlying cause of the IRD remaining unknown.

According to published data, the detection rate of causative variants in IRD patients determined by whole-genome sequencing (WGS) is approximately 10–18% higher than that achieved with panel sequencing across multiple cohort studies [8,11,12,13].

In a Russian cohort of patients with suspected IRDs, the diagnostic yield of panel sequencing (212 genes) was 46% [14]. Among patients without an established molecular diagnosis, 20% were found to carry variants of uncertain significance (VUS) or single pathogenic variants in genes associated with autosomal recessive IRDs. Thus, the aim of the present study is to evaluate the diagnostic effectivity of whole-genome sequencing in a cohort of patients carrying single heterozygous variants in autosomal recessive IRD-associated genes.

## 2. Results

### 2.1. Detected Variants Explaining the Cause of Diseases

**Figure 1:**
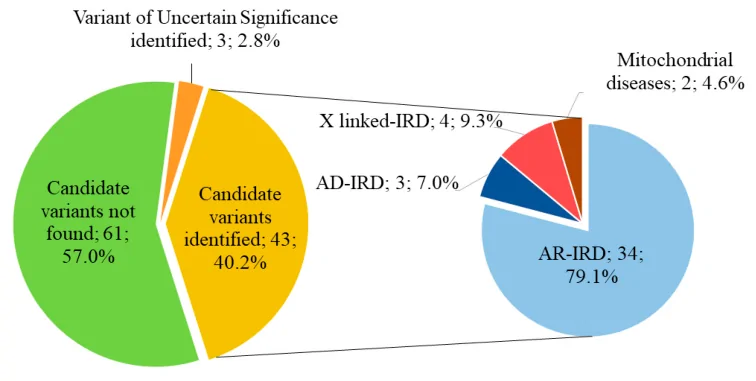
Results of whole-genome sequencing performed in 107 unrelated patients with retinal disorders. AR—autosomal recessive; AD—autosomal dominant.

During the analysis, candidate genetic variants associated with inherited retinal diseases were identified in 43 of 107 examined patients (40.2%) (Figure 1). The overall diagnostic yield was 40.2% (43/107), with 25.2% of cases (27/107) potentially resolved by second variants in target genes (*ABCA4, USH2A, CNGB3, EYS, CRB1,* and *CEP290*) and 15.0% (16/107) resolved by variants in alternative IRD genes including different inheritance patterns—autosomal recessive (*n* = 7), autosomal dominant (*n* = 3), X-linked (*n* = 4), and mitochondrial (*n* = 2).

#### 2.1.1. Spectrum of Variants Identified in Genes Associated with Common Autosomal Recessive Retinal Diseases

Among cases of autosomal recessive IRDs (*n* = 34), a second candidate genetic variant in the genes *ABCA4, USH2A, CNGB3, EYS, CRB1,* and *CEP290* was identified in 79.4% of patients (*n* = 27), whereas across the entire cohort of probands (*n* = 107), this proportion was 25.2%. Likely pathogenic variants in the *EYS* gene were detected in nine patients, followed by *USH2A* (*n* = 8), *ABCA4* (*n* = 5), *CNGB3* (*n* = 3), *CRB1* (*n* = 1), and *CEP290* (*n* = 1) (Table 1). In *EYS*, the majority of identified variants were represented by CNVs and structural rearrangements. In *ABCA4*, previously reported deep intronic variants were identified in three cases. In *USH2A*, a gross deletion encompassing exons 22–24 was detected in four patients. In *CNGB3*, two patients carried the same deletion of exon 15; in one patient, a structural rearrangement of the delINV type was identified on the second allele. One variant was detected in *CRB1* (a gross deletion of exon 8) and *CEP290* (a previously described deep intronic variant). Notably, a common deep intronic variant in *CEP290* had been previously included into the custom panel and is therefore not presented in the current analysis, although it is a frequent variant in the Russian population (allele frequency up to 25%) [15]. Overall, the spectrum of second-allele variants identified in these 27 patients comprised 14 gross deletions (51.8%), seven non-coding variants (26.0%), four structural rearrangements (14.8%), one gross duplication (3.7%), and one exonic point variant (3.7%) detected in *ABCA4*. The latter variant, c.5512del/p.(His1838Thrfs*13), had been previously missed during sequencing on the IonTorrent platform, likely due to its location within a poly-G region, which is prone to sequencing errors such as delins events.

#### 2.1.2. Spectrum of Variants Identified in Genes Associated with Other IRD Phenotypes

Although the WGS approach was primarily aimed at identifying a second variant in common genes associated with autosomal recessive IRDs, candidate genetic variants in genes linked to other IRD phenotypes were identified in 16 patients (37.2%) (Table 2). These variants were found in genes associated with autosomal recessive, autosomal dominant, X-linked, and mitochondrial patterns of inheritance. In addition, variants were detected in genes associated with syndromic forms of IRDs (*BBS5, BBS10*, *ALMS1* and *COL11A1*).

### 2.2. Characterization of Identified CNVs and Structural Rearrangements

#### 2.2.1. *EYS* Gene

In the *EYS* gene, a gross deletion partially affecting exon 19 (NC_000006.12:g.64877752_64886823del) was identified in four unrelated Russian patients and represents a recurrent variant within the studied cohort. The localization of the right breakpoint within the coding region of exon 19 results in a frameshift, which is likely to lead to transcript degradation via nonsense-mediated decay (NMD). In all patients carrying this variant, the second allele harbored loss-of-function (LoF) variants, and the clinical phenotype was consistent with retinitis pigmentosa. The most probable mechanism underlying this rearrangement is non-homologous end joining (NHEJ).

Among other CNVs in *EYS*, a heterozygous tandem duplication of exon 22 (~84.5 kb; NC_000006.12:g.64731274_64815790dup) was identified. Given that exon 22 encodes part of a laminin G-like domain of the protein, this rearrangement is expected to disrupt the structural organization of the peptide and likely to lead to NMD, as the exon length is not a multiple of three. The right breakpoint of the duplication maps to a LINE-repeat (*L1* family), whereas the left breakpoint does not contain repetitive elements. Therefore, the duplication likely arose via NHEJ mediated by microhomology of 1 bp. According to published data, pathogenic gross duplications involving one (e.g., exon 14 or exon 30) or multiple exons have been previously reported in *EYS* [16,17]. The variant identified in this study was classified as likely pathogenic.

**Figure 2:**
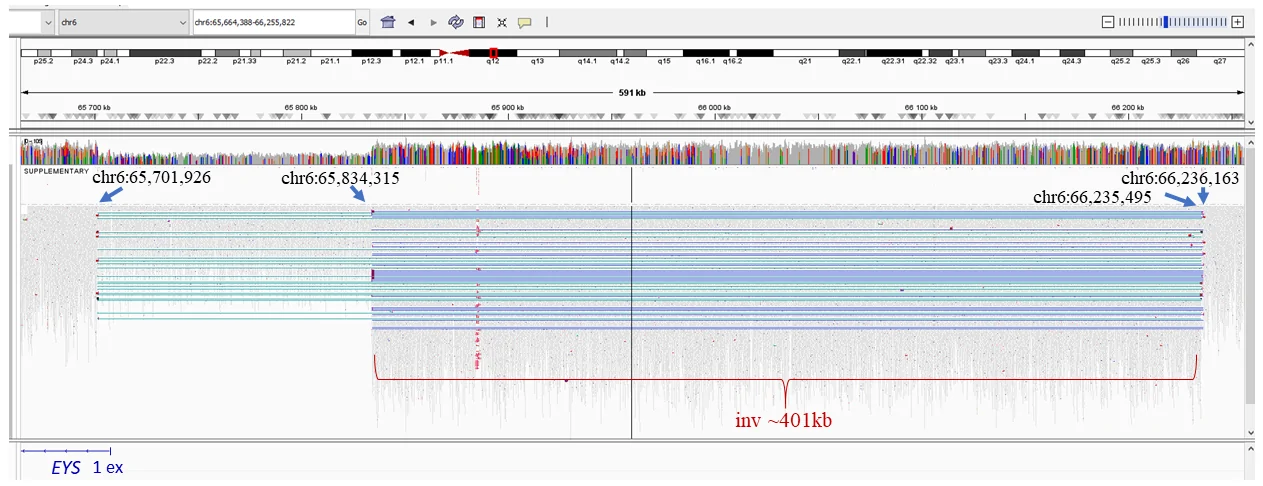
Schematic representation of the mapped breakpoints of a delINV structural rearrangement affecting the *EYS* gene in patient 14 (IGV screenshot).

All identified structural rearrangements affecting the *EYS* gene were unique. In patient 14, coverage analysis of sequencing data allowed to suggest the presence of a complex delINV rearrangement at locus 6q12 of approximately 534 kb in size (NC_000006.12:g.65701926_66236163delins65834315_66235495inv) (Figure 2). The first mapped breakpoint is located within intron 1 of *EYS* and is the start of a gross deletion (approximately 132 kb), encompassing exon 1 of the gene. The second breakpoint involves a 668 bp deletion that does not affect any annotated genes. Deletion of *EYS* exon 1 has been repeatedly reported in patients with retinal dystrophy and is classified as pathogenic [18,19]. Loss of the first non-coding exon is likely to disrupt transcription and result in the absence of protein production.

**Figure 3:**
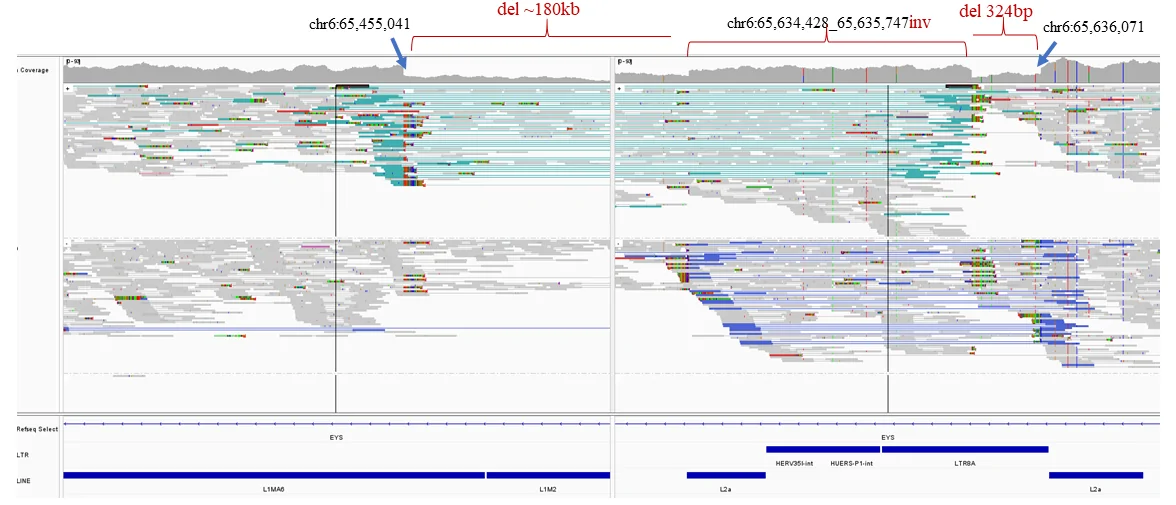
Mapped breakpoints of a delINV structural rearrangement affecting the *EYS* gene in patient 17 (IGV screenshot). Repeats are shown below.

**Figure 4:**
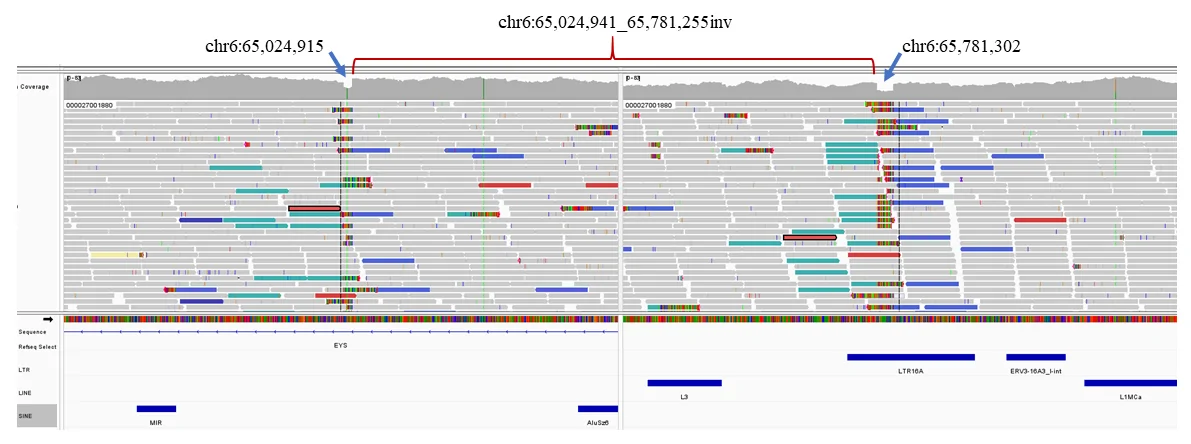
Mapped breakpoints of a delINV structural rearrangement affecting the *EYS* gene in patient 21 (IGV screenshot). Repeats are shown below.

In patient 17, a delINV rearrangement of approximately 181 kb was identified (NC_000006.12:g.65455041_65636071delins65634428_65635747inv) (Figure 3). The left breakpoint is located within intron 5 of *EYS*, which contains an *L2a*-repeat (LINE). At this breakpoint, a heterozygous deletion of approximately 179 kb was detected, affecting exons 3–5 of the gene. The right breakpoint was mapped to intron 2 within a region containing an *LTR8A* retrotransposon. This rearrangement most likely arose via NHEJ during the repair of two double-strand breaks, as the involved repetitive sequences are not highly homologous. In patient 21, a larger inversion (~756 kb) was identified (NC_000006.12:g.65024915_65781302delins65024941_65781255inv) (Figure 4), encompassing exons 1–13 of the *EYS* gene. One of the inversion breakpoints was located outside the gene, within a region containing an *LTR16A*-repeat. Such rearrangements, affecting functionally critical regions of the gene and leading to exon copy number loss, severely disrupt gene architecture and are highly likely to result in the production of a truncated, nonfunctional protein.

#### 2.2.2. *USH2A* Gene

In the *USH2A* gene, the same previously described gross deletion encompassing exons 22–24 (chr1:216086061_216149817del) was identified in four probands. This deletion does not result in a frameshift (p.(Gly1543_Pro1662del)). This deletion has been previously described in Russian patients with isolated hearing loss and Usher syndrome, but its exact boundaries could not be determined due to the use of MLPA [20]. The genomic coordinates of this deletion (63,757 bp size) are consistent with those reported in other studies [21,22]. Three patients (№№ 9–11) carrying this variant were referred with suspected isolated retinitis pigmentosa, whereas one patient (№ 12) was suspected of having Usher syndrome (childhood-onset hearing impairment). The deletion of exons 22–24 is not phenotype-specific for *USH2A*-associated phenotypes; however, gross deletions in combination with LoF variants on the second allele are generally associated with a more severe phenotype and earlier disease onset compared to genotypes harboring missense variants.

Additional CNVs identified in *USH2A* included deletions of exon 4 and exons 57–72. The deletion of exons 57–72 was detected in the heterozygous state. Both breakpoints of this deletion are located within LINE repetitive elements (*L2a* and *L1MA4A*), suggesting that non-allelic homologous recombination (NAHR) may have occurred between these regions. In addition, this ~324 kb deletion completely encompassed the *KCTD3* gene (NM_016121.4), in which biallelic variants have been associated with autosomal recessive epileptic encephalopathy and ciliopathy [23,24]. The large size of this deletion, affecting the C-terminal region of the usherin protein, is likely to result in the production of a truncated, nonfunctional protein lacking key Fibronectin type III domains, as well as transmembrane and PDZ1-binding domains.

Another gross deletion involving exon 4 (~54 kb) affects a functionally important region of the protein—the Laminin G-like domain. This deletion is predicted to produce a truncated transcript; seeing as the exon length is not a multiple of three, it is therefore likely to undergo NMD. Deletion of exon 4 has been previously reported in patients with retinitis pigmentosa and classified as pathogenic [25,26]. Sequence alignment at the breakpoint junctions revealed a 3 bp microhomology, suggesting a classical NHEJ mechanism. In the present study, patient 6 and patient 8 carrying deletions of exon 4 and exons 57–72 also have the variants c.13335_13347delinsCTTG/p.(Glu4445_Ser4449delinsAspLeu) and c.13396C>T/p.(Pro4466Ser), respectively. These probands did not exhibit hearing impairment.

#### 2.2.3. *CNGB3* Gene

In the present study, a heterozygous gross deletion of exon 15 in the *CNGB3* gene (chr8:86598578_86606871del) was identified in two unrelated probands with cone dystrophy. This deletion results in a frameshift and affects a conserved domain—the cGMP-binding site—essential for proper protein function. The left breakpoint of the deletion maps to an LTR-repeat of the *ERVL*-*MaLR* family, whereas the right breakpoint is not located at a repeat region. The rearrangement likely arose due to limited sequence similarity (2 bp microhomology) in the flanking regions via a NHEJ mechanism [27]. According to the gnomAD SVs v4 database, the allele frequency of this variant is 0.0016%. This deletion has also been previously reported by Mayer A.K. et al. in combination with another pathogenic LoF variant in a patient with achromatopsia [27].

**Figure 5:**
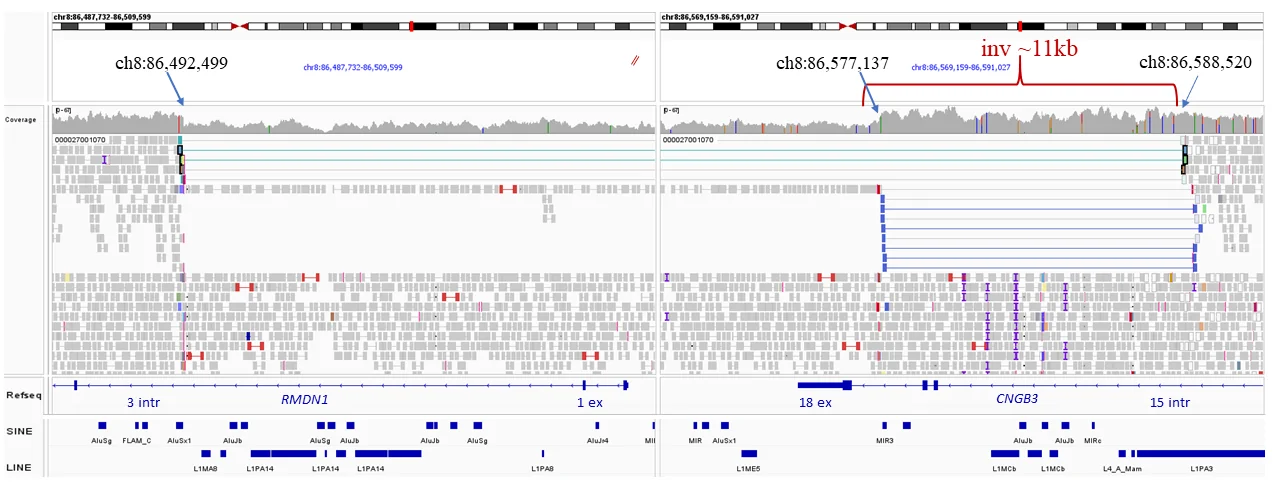
Visualization of the breakpoints of a delINV rearrangement involving the *CNGB3* gene in patient № 23 (IGV screenshot). Repeats are shown below.

In patient 23, prior panel-based sequencing identified a single variant, c.819_826del, in *CNGB3*. The proband (2 years old) presented with congenital nystagmus, and at the time of examination exhibited photophobia, impaired color vision, and reduced visual acuity—features consistent with cone dysfunction. Whole-genome sequencing (trio analysis) revealed a heterozygous delINV rearrangement at locus 8q21.3 (NC_000008.11:g.86492499_86588520delins86577137_86588520inv) of approximately 96 kb (Figure 5). The 3′ breakpoint (intron 15 of *CNGB3*) maps to a LINE-repeat of the *L1*-family (*L1PA3*), whereas the 5′ breakpoint overlaps an *Alu*-repeat (*AluSx1*). This rearrangement encompasses the *RMDN1* and *CPNE3* genes, which are not associated with any phenotype according to the OMIM database, as well as the *CNGB3* gene, in which exon 18 (the last exon) is deleted and exons 16–17 are inverted. The rearrangement was inherited from the mother, while the LoF variant was inherited from the father, thereby confirming the pathogenicity of the identified structural variant.

#### 2.2.4. *ABCA4* Gene

In the *ABCA4* gene, a gross deletion of exon 30 (~1 kb; chr1:94028926–94029978del) resulting in a frameshift was identified in proband 1. In patient 1 (16 years old), progressive bilateral visual acuity had been observed since the age of 9 years, with no significant family history. Ophthalmoscopy and instrumental examinations suggested Stargardt disease and the patient was subsequently referred for genetic testing. The identified deletion is absent from population frequency databases (gnomAD SVs v2) and is not reported in ClinVar. Sequence alignment at the breakpoint junctions revealed a 4 bp microhomology, suggesting a NHEJ mechanism. Although pathogenic CNVs have been described in *ABCA4*, they occur relatively less frequently (~9%) compared to deep intronic variants (~30%) as missing second alleles [28].

### 2.3. Complex Chromosomal Rearrangement Affecting the CWC27 Gene

**Figure 6:**
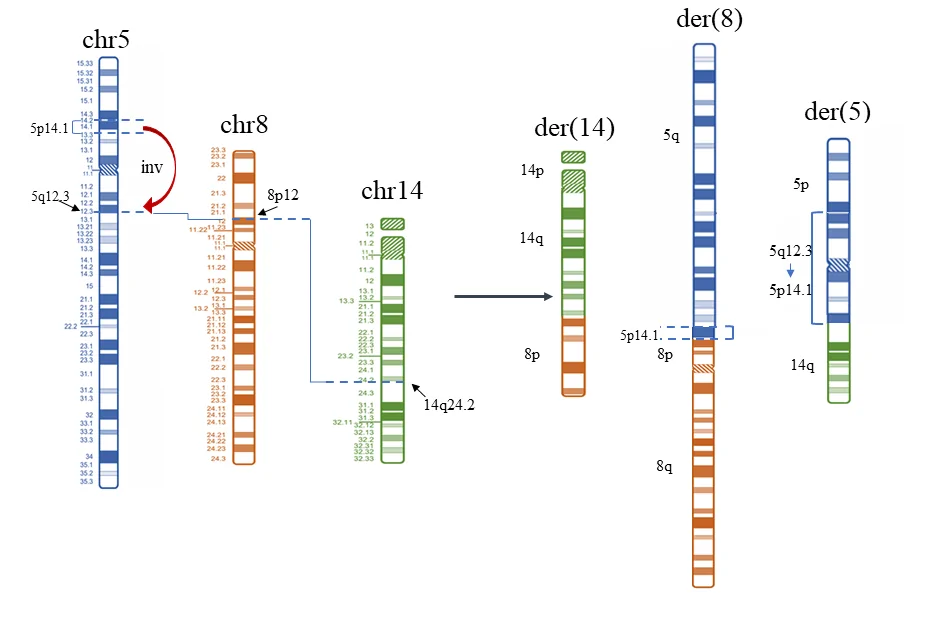
Schematic representation of derivative chromosomes resulting from a complex rearrangement involving chromosomes 5, 8, and 14 in patient 34. Dashed lines indicate the breakpoint positions.

**Figure 7:**
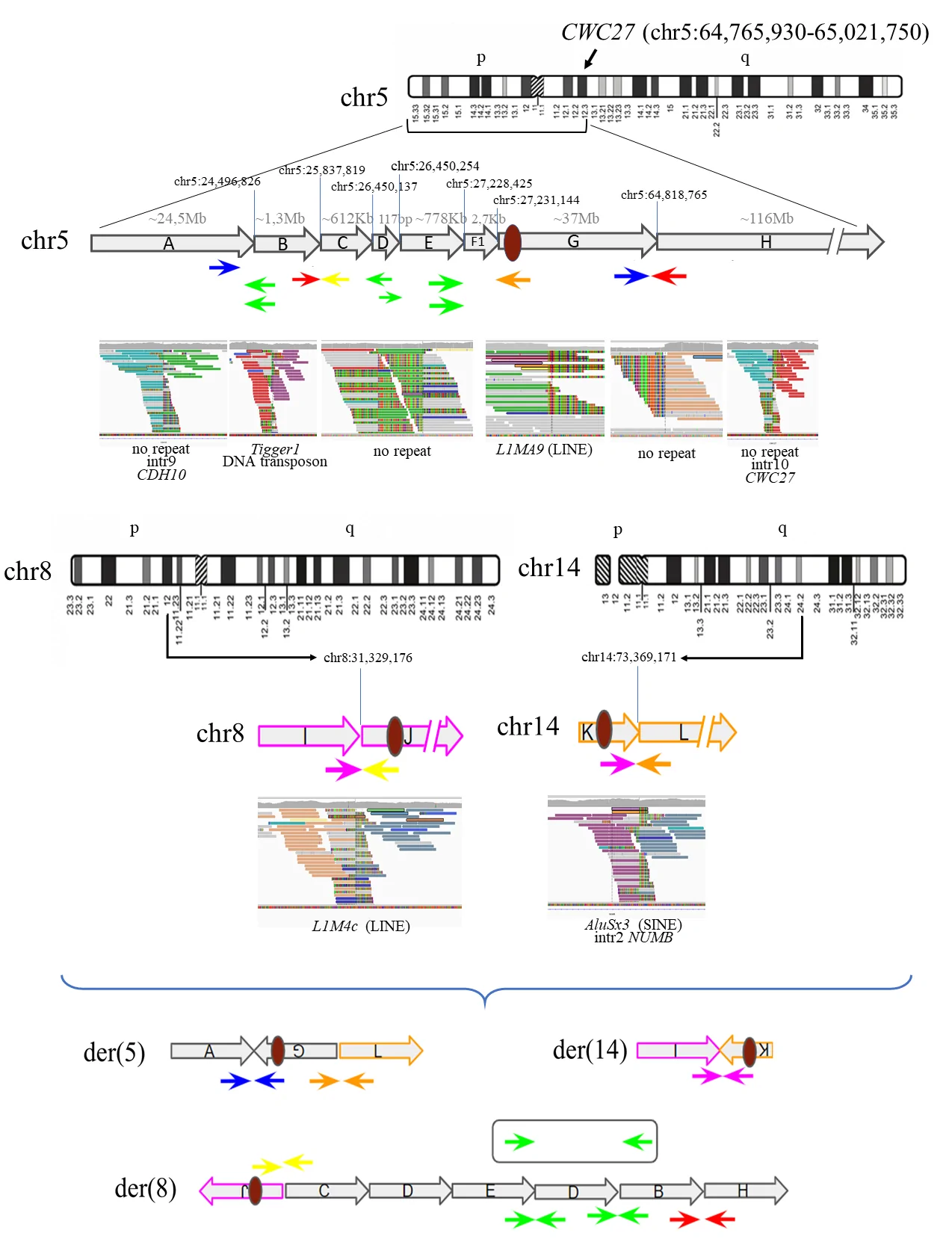
Schematic representation of the mapped breakpoints of a complex chromosomal rearrangement resulting in the formation of three derivative chromosomes in patient 34. Note: paired-end reads are shown in the same color in the “insert size and pair orientation” mode (IGV browser screenshots showing breakpoints at 1–2 kb scale). The centromere is marked by a brown oval.

In patient 34 (20 years old), analysis of WGS data revealed a heterozygous likely pathogenic LoF variant in the *CWC27* gene, c.1002dup/p.(Val335Serfs*13). Copy number analysis additionally identified a balanced complex chromosomal rearrangement involving chromosomes 5, 8, and 14 (Figure 6). In *CWC27*, located at locus 5q12.3, a breakpoint was detected within intron 10 (chr5:64818765), with paired-end reads mapping to chromosome 8. As a result of this rearrangement, three derivative chromosomes were formed. One of them, der(5), contained an inverted segment spanning 5p14.1 to 5q12.3, accompanied by loss of a portion of 5p14.1, which was translocated to chromosome 8 together with the long arm of chromosome 5 (Figure 6). Within the affected 5p14.1 region (chr5:24496827_27231144), multiple breakpoints were identified, leading to translocation of the terminal segment (fragment B in Figure 7), deletion of the pericentromeric region (fragment F1), and duplication of a small internal segment (fragment D). We hypothesize that this rearrangement most likely occurred de novo and resulted from chromothripsis. Given that the breakpoint disrupts the coding sequence of the gene and a second loss-of-function variant was identified, this rearrangement represents a candidate variant.

The breakpoints across the three chromosomes did not involve genes with established disease linkage, with the exception of *CWC27*, where the breakpoint occurred within intron 10. The *CWC27* gene (NM_005869.4), encoding a peptidyl-prolyl isomerase (PPIase), is involved in protein–protein interactions during the assembly and remodeling of spliceosomal components. The gene comprises 14 exons encoding a 427-amino-acid protein that contains an N-terminal proline-binding domain, which is critical for its function. Experimental studies have demonstrated that *CWC27* acts as a splicing factor; knockout Cwc27^K338fs^/^K338fs^ mice exhibit a significant increase in transcripts with retained introns and alternative exons compared to controls [29]. Given the sensitivity of the retina to cellular stress, increased expression of genes involved in inflammatory responses may represent a key driver of early degenerative processes.

Biallelic pathogenic variants in the *CWC27* gene have been reported in patients with both isolated and syndromic forms of retinal dystrophy. In addition to ocular manifestations, affected individuals may present with developmental delay (both physical and intellectual), skeletal, craniofacial, and ectodermal anomalies, as well as neurological impairments. To date, no clear genotype–phenotype correlation has been established. In patient 34 from the present study, medical history indicated that reduced visual acuity in both eyes was first noted by the parents at the age of 3 years, followed by impaired night vision at the age of 5 years. A rod–cone retinal dystrophy was suspected by an ophthalmologist. At the age of 14 years, the patient underwent surgical correction for severe (grade IV) scoliosis. Additional phenotypic features included a low anterior hairline, a high-arched palate, and torticollis. Given the data described above, we suggest that both variants in the *CWC27* gene may be candidate variants associated with the patient’s phenotype. However, validation of the chromosomal rearrangement by reference methods (such as karyotyping, fluorescence in situ hybridization, or Optical Genome Mapping) must be performed. Additional confirmatory testing was recommended for the patient and his parents.

### 2.4. Structural Rearrangement Affecting the RP2 Gene

**Figure 8:**
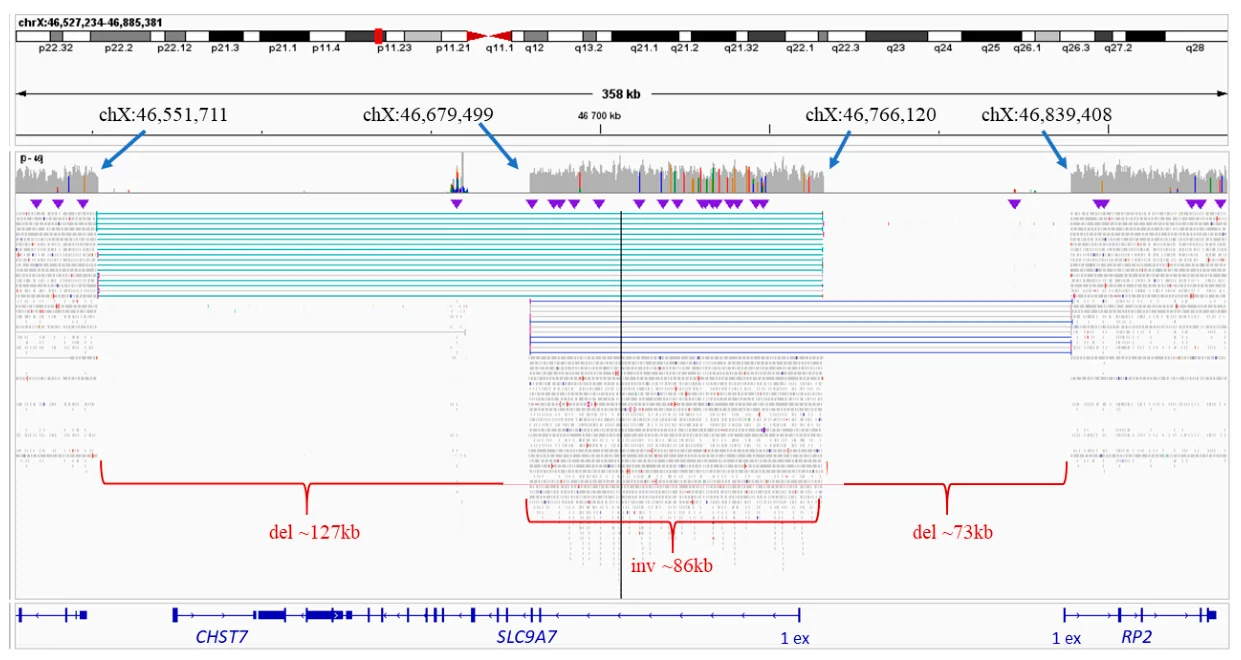
Visualization of the breakpoints of a delINV rearrangement involving the *RP2*, *SLC9A7*, and *CHST7* genes in patient 40 (IGV screenshot).

WGS identified a delINV rearrangement at locus Xp11.3 of approximately 287 kb in a hemizygous state in patient 40 (15 years old) (NC_000023.11:g.46551711_46839408delins46679500_46766119inv) (Figure 8). According to the medical history, the proband exhibited reduced visual acuity and impaired color vision from early childhood. Electrophysiological examination revealed a decreased b-wave amplitude on full-field electroretinography (ERG), and optical coherence tomography (OCT) demonstrated pronounced changes at the level of the retinal pigment epithelium and photoreceptor layer. Additionally, further anamnesis analysis indicated delayed speech and cognitive development, with the patient achieving single-word speech at approximately 3.5 years of age. The identified rearrangement resulted in the deletion of exon 1 of the *RP2* gene, which is associated with X-linked retinitis pigmentosa type 2. In addition, another deleted segment encompassed the entire *CHST7* gene (NM_019886.4), which is not currently associated with any phenotype according to OMIM, and exons 4–17 of the *SLC9A7* gene (NM_032591.3). Hemizygous variants in *SLC9A7* have been reported in patients with intellectual developmental disorder (OMIM:301024). A five-generational family has previously been described in which a severe retinitis pigmentosa phenotype co-segregated with mild-to-moderate intellectual disability, following an X-linked recessive pattern of inheritance [30]. This phenotype was attributed to a 1.27 Mb deletion at Xp11.2 affecting exons 1–3 of *RP2* as well as the *CHST7* and *SLC9A7* genes. Thus, WGS enabled identification of the genetic basis not only of the retinal pathology but also of the probable cause of the patient’s neurodevelopmental delay.

### 2.5. Variants Identified in Non-Coding Regions

In the present study, in three patients, three previously described *ABCA4* intronic variants were identified: c.4539+2064C>T, c.6817-679C>A, and c.859-546G>A, located in introns 30, 49, and 7, respectively. Functional studies conducted by other authors have demonstrated the pathogenic impact of these nucleotide substitutions on pre-mRNA splicing. The c.4539+2064C>T variant is relatively common in European populations [31], whereas c.6817-679C>A and c.859-546G>A are less frequent, according to the literature. In patient 7 (57 years old) with retinitis pigmentosa, a previously described pathogenic intronic variant, c.7595-2144A>G, was identified in the *USH2A* gene. This variant is most commonly observed in European patients with Usher syndrome type 2 [32]. Functional studies have shown that it leads to the inclusion of a 152 bp pseudoexon, resulting in p.(Lys2532Thrfs*56) [33]. Given that the C-terminal region of the protein is critical for protein–protein interactions, the resulting truncated protein is expected to be nonfunctional. Notably, this patient did not exhibit hearing impairment, and the onset of visual symptoms occurred at 18 years of age. On the second allele, the variant p.(Glu4445_Ser4449delinsAspLeu) was identified, which has been previously shown to be associated with non-syndromic retinitis pigmentosa [14].

In four patients (№№ 13, 22, 27, and 41), novel intronic variants were identified in *USH2A*, *EYS*, and *CEP290* genes, associated with autosomal recessive retinal diseases, and in the *CHM* gene, responsible for X-linked choroideremia. For all variants, the SpliceAI algorithm predicted splicing alterations; therefore, these variants were subjected to in vitro functional analysis by minigene assay.

Minigenes with wild-type or mutant fragments of *CEP290, CHM, EYS*, and *USH2A* were generated as described in the Materials and Methods section. HEK293T cells were transfected with minigenes and, two days after, cells were collected for the RNA analysis.

**Figure 9:**
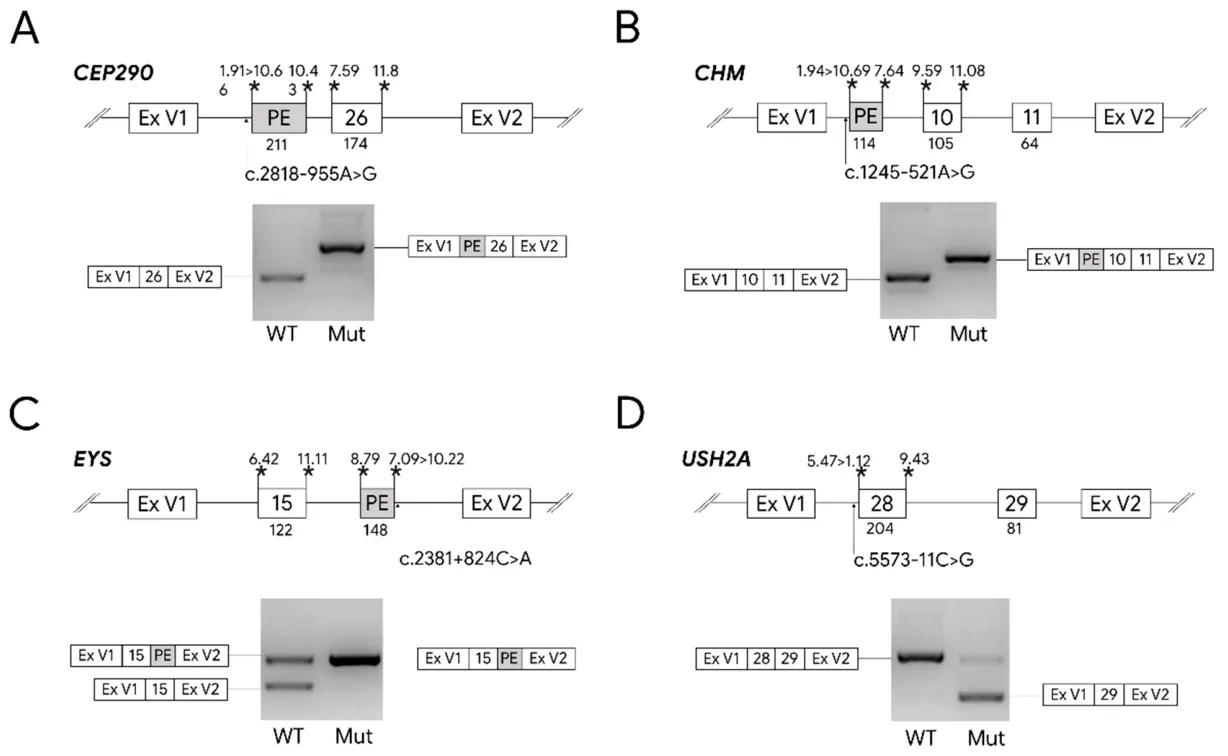
Results of the RNA analysis for the studied variants in the *CEP290* (**A**), *CHM* (**B**), *EYS* (**C**), and *USH2A* (**D**) genes. Schemes of the minigenes and visualization of minigene-specific splicing products by electrophoresis in a 3% agarose gel are shown. Ex V1 and Ex V2—exons of the minigene vector; PE indicates a pseudoexon. Asterisks (*) indicate splice sites and their predicted strength (MaxEntScan score).

The *CEP290*:c.2818-955A>G variant was predicted to create a strong acceptor splice site (SpliceAI AG 0.99; MES (MaxEntScan score) 1.91 > 10.66). Minigene assay demonstrated that the variant leads to the inclusion of a 211 bp pseudoexon (r.2817_2818ins211; p.(Glu940Leufs*15)) with no residual amount of the correct transcript (Figure 9A). The c.2818-955A>G variant was identified in a compound heterozygous state with c.1334_1339del; p.(Leu445_Lys446del) in patient 27 (42 years old), who first complained of visual impairment at the age of 15 and was suspected of having retinitis pigmentosa by the age of 20. No additional systemic alterations were observed. The likely pathogenic *CEP290* variant c.1334_1339del; p.(Leu445_Lys446del), resulting in an in-frame deletion, has not been previously described but is present in the gnomAD database (v3.1.2) at a frequency of 0.0007%. This variant is located within a highly conserved region of a coiled-coil domain, which is critical for maintaining the structural stability of the protein. It is likely to represent a "moderate" allele that may affect local protein folding and disrupt protein–protein interactions. In combination with the “severe” intronic variant c.2818-955A>G, it likely results in a milder phenotype with later disease onset compared to the early-onset vision loss characteristic of Leber congenital amaurosis.

The *CHM*:c.1245-521A>G variant was predicted to create a strong acceptor splice site (SpliceAI AG 0.82; MES 1.94 > 10.69). Minigene assay demonstrated that the variant leads to the inclusion of a 114 bp pseudoexon (r.1244_1245ins114; p.(Cys416_Glu653delinsHisTyrProThrArgAspHisIleAlaIleLeuGlyGlnLysArgAsnGlnHisLeuThrGluLys*)) with no residual amount of the correct transcript (Figure 9B). *CHM*:c.1245-521A>G was identified in a hemizygous state in patient 41 (33 years old) with reduced distance vision and impaired low-light orientation were noted from adolescence. Over the past seven years, progressive visual deterioration was observed. At the time of examination, marked constriction of visual fields was documented. Fundoscopic evaluation revealed choriocapillaris atrophy with a prominent vascular pattern due to diffuse degeneration, along with peripheral bone spicule pigmentation. No family history of visual impairment was reported. Taken together, the molecular and clinical findings indicate that the identified intronic variant in *CHM* is the most likely cause of the disease.

The *EYS*:c.2381+824C>A variant was predicted to create a strong donor splice site (SpliceAI DG 0.57; MES 7.09>10.22). Minigene assay demonstrated that the variant leads to the activation of a 148 bp pseudoexon (r.2381_2382ins148; p.(Cys795Glufs*51)) (Figure 9C). A faint pseudoexon-containing product was also detected from the wild-type EYS minigene; the mutant construct produced a substantially higher proportion of this product than the isogenic WT construct. The c.2381+824C>A variant was identified in the compound heterozygous state with frameshift variant c.8648_8655del/p.(Thr2883Lysfs*4) in a 35-year-old patient who had clinical signs of retinitis pigmentosa (peripheral visual field constriction, night blindness, decreased visual acuity for distance, with complaints since age 30).

The *USH2A*:c.5573-11C>G variant was predicted to weaken the acceptor splice site of the exon 28 (SpliceAI AL 0.31; MES 5.47 > 1.12). Minigene assay demonstrated that the variant leads to the skipping of exon 28 and some residual amount of correct transcript (Figure 9D). The exon skipping results in an in-frame deletion of 68 amino acids (r.5573del204; p.(Gly1858_Thr1925del)) within the functionally important Fibronectin type III domain of the usherin protein. This domain is known to be a hotspot for pathogenic missense variants, and a similar intronic variant, c.5573-19A>G, has previously been reported to cause exon 28 skipping [22,32], supporting the critical role of Fibronectin type III domains in maintaining protein structure and mediating protein–protein interactions [34]. The c.5573-11C>G variant was identified in the compound heterozygous state with the nonsense variant c.4174G>T/p.(Gly1392*) in a patient with retinitis pigmentosa who did not exhibit hearing impairment at the time of examination.

### 2.6. Identified Variants in Genes Associated with Syndromic Forms of IRDs

Among autosomal recessive syndromic IRDs, three patients were found to carry exonic point variants in the *BBS5, BBS10*, and *ALMS1* genes, associated with Bardet–Biedl syndrome types 5 (OMIM:615983) and 10 (OMIM:615987), and Alstrom syndrome (OMIM:203800), respectively. Three LoF variants were identified, two of which were detected in the homozygous state in Russian patients for the first time, along with one previously described missense variant in *BBS10*. According to clinical records, patient 28 (11 years old), with a hypersthenic body habitus, had undergone surgical correction of bilateral polydactyly of the feet in early childhood and exhibited delayed psychomotor and speech development. In cases 29 (20 years old) and 30 (10 years old), limited clinical information was available beyond primary visual complaints (congenital nystagmus, reduced visual acuity, photophobia), with preliminary diagnoses of rod–cone dystrophy and central retinal dystrophy, respectively. These molecular genetic findings require careful comparison with the clinical phenotype.

In two probands (№№ 36 and 37), the likely cause of retinal dystrophy was associated with variants in the *COL11A1* gene. Patient 36 presented with concentric constriction of visual fields in both eyes, with non-recordable rod responses on electroretinography, while fundoscopic examination did not reveal pronounced alterations. Patient 37 reported impaired night vision since childhood and peripheral visual field constriction; fundoscopic examination revealed multiple dystrophic lesions along the vascular arcades and peripheral bone spicule pigmentation. At the age of 29, this patient underwent bilateral cataract surgery. No additional information regarding craniofacial features or systemic involvement was available for either proband. Heterozygous variants in *COL11A1* have been previously associated with Stickler syndrome type 2 (OMIM:604841) and Marshall syndrome (OMIM:154780). The mutational spectrum of this gene is predominantly composed of variants affecting splicing. In the present study, two novel variants were identified: a single-nucleotide deletion at the splice donor site of intron 30 (c.2502+1del) and a 43 bp delins affecting the canonical splice acceptor site of intron 64 (c.4972-42_4972delinsTTTATA). Both variants were detected in the heterozygous state and classified as likely pathogenic according to ACMG. These findings require further comparison with the clinical phenotype, as atypical cases of Stickler syndrome with predominantly ocular manifestations and minimal systemic involvement have been described in the literature [35]. It is also possible that the observed retinal degeneration represents a secondary manifestation at the time of examination.

### 2.7. Variants in Genes Associated with Mitochondrial Retinopathies

An important advantage of WGS is its ability to detect variants in mitochondrial DNA (mtDNA). In the present study, two probands (№№ 42 and 43) were found to carry previously reported pathogenic variants in the *MT-ATP6* (chrM:8993T>G) and *MT-ND6* (chrM:14484T>C) genes. The former was detected at 84% heteroplasmy, whereas the latter was present in a homoplasmic state (100%). The m.14484T>C variant in *MT-ND6* is a well-established cause of Leber hereditary optic neuropathy (LHON). It should be noted that optic nerve atrophy may also occur as a secondary manifestation of other primary retinal disorders (e.g., retinitis pigmentosa), which can complicate differential diagnosis at advanced disease stages.

The variant m.8993T>G in *MT-ATP6* has been reported in patients with Leigh syndrome (OMIM:500017) and NARP syndrome (neuropathy, ataxia, and retinitis pigmentosa; OMIM:551500). It is known that individuals harboring this variant at heteroplasmy levels below 60% are typically asymptomatic or exhibit only mild features such as retinitis pigmentosa, whereas heteroplasmy levels between 75% and 90% are associated with NARP, and levels above 90% with Leigh syndrome. In patient 42 (35 years old) carrying the *MT-ATP6* variant, no systemic manifestations were reported apart from visual impairment. Ophthalmological examination revealed peripheral bone spicule pigmentation, macular dystrophic changes, and a waxy optic disc. According to the medical history, the patient’s child died in early childhood with a preliminary diagnosis of West syndrome. Possibly a higher heteroplasmy level of the same variant contributed to the severe phenotype observed in the child. Therefore, in cases where mtDNA variants are identified, it is recommended to assess heteroplasmy levels in multiple tissues (e.g., blood and urinary sediment) in both the proband and maternally related family members, as well as to carefully compare genetic findings with the clinical presentation.

## 3. Discussion

Due to the pronounced clinical and genetic heterogeneity of inherited retinal diseases, the application of mass parallel sequencing technologies is the preferred approach for determining the molecular cause of the disorder. The diagnostic effectiveness of targeted sequencing varies widely (30–75%) depending on the studied cohorts [36,37,38,39]. At present, WGS offers substantial advantages over gene panels and WES, as it provides more uniform and complete genome coverage, encompassing both coding and non-coding regions. The detection rate of causative variants in IRD patients using WGS exceeds that of targeted enrichment methods by approximately 15–20% [8], primarily due to the identification of CNVs, complex structural rearrangements, deep intronic variants, and variants in the genes not included in targeted sequencing.

In the present study, a Russian cohort of 107 patients was analyzed, all of whom carried single heterozygous variants in the *ABCA4, USH2A, EYS, CRB1, RPE65, CNGB3, CNGA3, CEP290,* and *GUCY2D* genes associated with autosomal recessive retinal diseases. Among them, second pathogenic or likely pathogenic variants were identified in 27 patients (25.2%) in six genes (*ABCA4, USH2A, EYS, CRB1, CNGB3,* and *CEP290*). The detected variant spectrum included eight different CNVs (seven gross deletions and one duplication), seven non-coding variants, four structural rearrangements of the delINV type, and one point variant in exon 39 of the *ABCA4*.

No second variants were identified in patients carrying single pathogenic variants in the *RPE65, CNGA3,* and *GUCY2D* genes. This suggests that WGS has limited utility for detecting additional variants in these genes, as their mutation spectra are predominantly composed of coding-region point variants, including those affecting canonical splice sites [40].

An important advantage of WGS is its ability to detect structural variants, including CNVs, gross inversions, and translocations. In this study, such variants were identified in 24 of 43 patients with an established molecular diagnosis. Structural rearrangements often arise as a result of NAHR between repetitive elements flanking unique homologous sequences. For example, in the *USH2A* gene, a deletion encompassing exons 57–72 was identified, with a likely mechanism involving NAHR mediated by LINE-repeats. In contrast, rearrangements lacking flanking repetitive elements are more likely to result from NHEJ.

In the present study, three delINV rearrangements were identified in the *EYS* gene, one in *CNGB3*, one in *RP1*, and one complex rearrangement involving three chromosomes, one of which affected the *CWC27* gene associated with syndromic retinitis pigmentosa. This rearrangement, described here for the first time in *CWC27*, represents a rare class of structural variant. Such changes should be interpreted with caution, since short-read WGS may result in incorrect mapping of breakpoints, especially in repeat-rich regions. The identified rearrangement requires validation by alternative methods. This case highlights the importance of considering chromosomal changes in ophthalmological practice.

In this cohort, structural variants were most frequently observed in the *EYS* gene. A recurrent deletion partially affecting exon 19 was identified in four patients and was not previously reported at such a high allele frequency in any cohort study. In the Russian population, this variant accounted for 9.5% of all known pathogenic alleles in a group of patients with *EYS*-associated retinal diseases. This deletion was first described by Perez-Carro et al. (2016) in a compound heterozygous state with a LoF variant in a patient with retinitis pigmentosa; however, its precise breakpoints were not determined [41]. To date, no other studies have reported deletion of exon 19, although in the gnomAD SVs v4 database (accessed 10 May 2026), its allele frequency is estimated at 0.06% (0.1% in the European population). Its apparent underrepresentation in the literature may be attributed to technical limitations of targeted sequencing approaches, which often do not allow us to determine precise deletion breakpoints.

In the study by Van Schil K. et al. (2018) [42], the analysis of IRD-associated genes and all reported CNVs revealed a correlation between gene size and the number of repetitive elements (LINE, SINE, *Alu*). Notably, the *EYS* and *USH2A* genes were shown to be particularly susceptible to structural rearrangements [42].

In the *USH2A* gene, the deletion of exons 22–24 represents a recurrent variant among Russian patients. This CNV has also been reported in individuals with Usher syndrome type II and retinitis pigmentosa from Bulgaria [43], Italy [44], the United Kingdom [8], Denmark [45], and Germany [22]. However, in contrast to the present study, it was typically observed in no more than one or two cases per cohort. The relatively high frequency of this deletion in both European and Russian populations suggests that it likely arose from an ancestral mutational event.

It is known that CNVs in the *CNGB3* gene account for approximately 2% of all detected variants, with structural rearrangements representing more than one-third of previously "missing" variants [27]. In the present study, the same exon 15 deletion in *CNGB3* was identified in two patients, emphasizing the importance of searching for such variants in individuals with cone dystrophy. In another gene, *PRPF31*, associated with autosomal dominant retinitis pigmentosa, CNVs are reported more frequently than in other IRD-associated genes. In this study, one patient was found to carry a gross deletion encompassing exon 1, the 5′-UTR region of *PRPF31*, and the adjacent *TFPT* gene (transcription factor 3-interacting protein). Similar heterozygous deletions have previously been described in families with retinitis pigmentosa [46]. The genomic region encompassing these genes is enriched in *Alu*-repetitive elements, rendering it unstable and prone to unequal recombination events.

In the present study, DIVs were identified in seven out of 43 solved cases, accounting for 16.3%. Three previously reported DIVs were detected in the *ABCA4* gene (c.4539+2064C>T, c.6817-679C>A, c.859-546G>A), supporting the existing evidence of the high prevalence of such variants in this gene [11,28]. In the *USH2A* gene, one patient carried the common European pathogenic variant c.7595-2144A>G [47]. In three patients, novel DIVs were identified in the *EYS* (c.2381+824C>A), *CEP290* (c.2818-955A>G), and *CHM* (c.1245-521A>G) genes. Functional analysis using minigene assays demonstrated that these variants affect normal splicing by inducing pseudoexon inclusion, resulting in frameshift alterations of the mRNA. Given some pseudoexon inclusion from the WT minigene, the assay supports rather than confirms splice disruption by c.2381+824C>A. Another intronic variant in *USH2A*, c.5573-11C>G, was shown to cause skipping of exon 28 with some residual amount of the correct transcript, leading to an in-frame deletion. This alteration is likely to affect protein structure, as it involves the functionally important fibronectin type III domain. However, for a more accurate interpretation of this variant at the protein level, additional studies are needed, particularly the evaluation of expression levels directly in retinal organoids. Thus, WGS combined with functional evaluation of variants predicted to affect splicing can substantially improve the diagnostic yield in IRD cases. The identified variants may also represent promising therapeutic targets for emerging approaches, including antisense oligonucleotide-based therapies and CRISPR/Cas9 genome editing [48].

In addition, variants were identified in genes associated with syndromic retinal disorders (*BBS5, BBS10, ALMS1, COL11A1*) as well as in genes linked to mitochondrial retinopathies (*MT-ATP6, MT-ND6*). However, comprehensive data on extraocular manifestations were not available for all patients in this study, underscoring the importance of a full clinical assessment, including detailed personal and family history, to enable accurate differential diagnoses between isolated and syndromic forms of disease. It should also be considered that variants in the same gene may be associated with both syndromic and non-syndromic IRDs, as has been reported for BBS genes [49].

A definitive molecular diagnosis could not be established in 64 of 107 patients (60%). It cannot be excluded that these individuals are incidental carriers of variants in common genes associated with autosomal recessive IRDs, while the true cause of disease is different. To test this hypothesis, a comparative analysis of carrier frequencies for pathogenic/likely pathogenic variants was performed between the patient cohort (*n* = 1538 unrelated probands) and a control group of presumably healthy individuals (*n* = 4151). In the control group, 359 individuals were carriers of pathogenic variants in genes associated with autosomal recessive IRDs, corresponding to 8.6%. Based on this, the expected number of heterozygous carriers in the patient cohort would be 132 individuals. However, the observed number of heterozygotes among the 1538 patients was 186 (12.1%). Due to limited availability of biological material, WGS was performed in only 107 of these patients, of whom 43 (40.2%) had candidate variants identified. Given that approximately 90% of the 186 patients carried variants in common IRD genes (*ABCA4, USH2A, EYS, CRB1, RPE65, CNGB3, CNGA3, CEP290* and *GUCY2D*), the WGS results can be extrapolated to the entire cohort. Accordingly, if WGS had been performed in all 186 individuals, a molecular diagnosis would be expected in approximately 74 patients. Under this assumption, the number of true carriers in the cohort would be 112 (7.3%), which showed only a borderline, non-significant difference from the expected frequency of 8.6% (χ^2^ = 2.76, *p* = 0.097), providing no strong evidence for an excess of missed second variants in the target genes. Therefore, in the remaining unsolved patients, the single variants in these frequent genes are unlikely to be the cause of the disease. The true genetic etiology in these cases is likely attributable to variants in genes associated with autosomal dominant or X-linked IRD phenotypes. However, this does not exclude the possibility that some second variants in frequent AR genes were missed due to the limitations of short-read sequencing, or that the disease is caused by rare events such as digenic inheritance. Furthermore, the high prevalence of missense variants of uncertain significance in such genes may complicate molecular diagnosis, requiring additional functional studies and segregation analysis.

## 4. Materials and Methods

### 4.1. Cohort Characteristics

The study cohort comprised 107 unrelated patients with suspected inherited retinal diseases, including retinitis pigmentosa, Leber congenital amaurosis, cone dystrophy/achromatopsia, Stargardt disease, and other forms. Patients from various regions of Russia were referred to the Federal Research Centre for Medical Genetics between 2021 and 2023 for molecular confirmation of their clinical diagnose. All 107 patients had previously undergone genetic testing using a custom “Ophthalmo” gene panel comprising 212 genes (Supplementary S1). Sequencing was carried out on Ion Torrent platforms (PGM/S5; Thermo Fisher Scientific, Waltham, MA, USA) or Illumina MiSeq/NextSeq systems (Illumina, San Diego, CA, USA). The “Ophthalmo” panel spans 822,786 bp and includes 4423 amplicons ranging in size from 125 to 275 bp. The estimated coverage of coding regions, according to the Ion AmpliSeq Designer software, version 5.62, is 97.73%.

Written informed consent was obtained from all adult participants and from the legal representatives of patients under 18 years of age. The study was conducted in accordance with the principles of the Declaration of Helsinki and was approved by the Ethics Committee of the Research Centre for Medical Genetics.

### 4.2. Whole-Genome Sequencing

For whole-genome sequencing (WGS), we selected archived DNA samples isolated from peripheral blood leukocytes using the QIAamp DNA Mini Kit (Qiagen, Hilden, Germany) according to the manufacturer’s protocol. WGS was carried out at OOO "Biotek Kampus" using the DNBSEQ-T7RS platform. Extracted DNA was quantified using the Qubit™ dsDNA Quantification Assay Kit (Thermo Fisher Scientific, USA). For each sample, 1000 ng of genomic DNA (gDNA) was used for library preparation with the MGIEasy Fast PCR-Free FS DNA Library Prep Set V2.0 (MGI, Shenzhen, China), following the manufacturer’s instructions. DNA was fragmented by enzymatic fragmentation using magnetic beads. End repair and adapter ligation were performed using the MGIEasy UDB PF Adapters-96 Kit (MGI, Shenzhen, China). Library size distribution was assessed using the 4200 TapeStation system with Agilent D1000 ScreenTape (Agilent, Santa Clara, CA, USA). The PCR products were circularized and 75 fmol of ssCirDNA were amplified using rolling-circle amplification to generate DNA nanoball-based libraries, which were loaded onto a DNBSEQ-T7RS Sequencing flow cell with a DNBSEQ-T7RS High-throughput Sequencing Kit (MGI, Shenzhen, China). Sequencing was carried out on the DNBSEQ-T7RS platform (MGI, Shenzhen, China) with paired-end 150 bp reads with a minimum average genome coverage of 30x.

### 4.3. Exclusion of Related Individuals

KING v2.2.4 [50] was used to detect pairs of individuals related up to the third degree, applying a kinship coefficient threshold of 0.0442. This analysis was performed using post-QC WGS data. In all downstream analyses, only unrelated individuals were included, i.e., those more distantly related than third-degree relatives.

### 4.4. Analysis and Interpretation of Nucleotide Sequence Variants

Variant nomenclature was used in accordance with the recommendations of the Human Genome Variation Society (HGVS; version 21.1.3, https://hgvs-nomenclature.org). All identified variants were mapped to the human reference genome GRCh38/hg38. Sequencing data processing was performed using the “NGS-data-Genome” software (https://ngs-data-ccu.epigenetic.ru/main/, accessed on 1 July 2026), developed by the Bioinformatics Department of the Research Centre for Medical Genetics (registration № 2021662113). Visualization of nucleotide variants was carried out using Integrative Genomics Viewer (IGV, Broad Institute, Cambridge, MA, USA), version 2.19.1 (https://igv.org/).

Population allele frequencies of the identified variants were assessed using the Genome Aggregation Database (gnomAD v3.1.2) and the gnomAD Structural Variant Database (gnomAD SVs v4) (http://gnomad.broadinstitute.org/). Clinical relevance was evaluated using the Online Mendelian Inheritance in Man (OMIM) database (http://omim.org), ClinVar (https://www.ncbi.nlm.nih.gov/clinvar/ accessed on 1 July 2026), and the Leiden Open Variation Database (LOVD; www.lovd.nl/, accessed on 1 July 2026), as well as the scientific literature data. Variant pathogenicity was assessed using in silico tools VarSome, version 13.17, (https://varsome.com/) and Franklin, version 94.0 (https://franklin.genoox.com). The potential impact on splicing was evaluated using SpliceAI (https://spliceailookup.broadinstitute.org/). Classification of variant pathogenicity, including single-nucleotide variants and CNVs, was performed in accordance with the American College of Medical Genetics and Genomics (ACMG) guidelines [51,52]. For genes affected by the CNVs, dosage sensitivity was assessed using the ClinGen Dosage Sensitivity Map. For balanced SVs, an adapted approach based on the general ACMG principles was applied, considering the genes disrupted at the breakpoints and the available literature data. Only variants classified as pathogenic (P) or likely pathogenic (LP) were considered candidate genetic variants for molecular diagnosis.

During the primary analysis of genetic variants, priority was given to examining structural rearrangements, CNVs and non-coding variants in genes harboring a single previously identified pathogenic variant, with the aim of detecting the second "missing" allele. If no additional variant was identified on the second allele, an extended search for causative variants was performed across all known genes associated with inherited retinal diseases. Structural rearrangements were detected using the Manta algorithm [53], while copy number variants (CNVs) were identified using the Canvas tool (https://github.com/Illumina/canvas, accessed on 1 July 2026). To support SpliceAI results splice site strength was also calculated using MaxEntScan [54].

Validation of identified variants and determination of their *cis-/trans-* configuration in cases of autosomal recessive IRDs were performed by Sanger sequencing in some families. Sequencing was carried out on an ABI Prism 3500xl Genetic Analyzer (Thermo Fisher Scientific, USA) using the BigDye Terminator v1.1 Cycle Sequencing Kit (Thermo Fisher Scientific, USA), in accordance with the manufacturer’s protocol.

### 4.5. Functional Analysis

Minigene construct generation: DNA fragments encompassing the studied exon or predicted pseudoexon with a minimum of 200 flanking nucleotides were amplified using the Phanta Max Super-Fidelity DNA Polymerase (Vazyme Biotech Co., Ltd., Nanjing, Jiangsu, China) and cloned into the pSPL3_Flu2_mTK minigene vector (Bychkov et al., 2022) [55] at the EcoRV restriction site using the ClonExpress Ultra One Step Cloning Kit V3 (Vazyme Biotech Co., Ltd., China). Both wild-type and mutant minigene constructs were verified by Sanger sequencing. Oligonucleotide primers used in the functional assay are listed in Supplementary S2.

Cell culture and transfection: HEK293T cells (ATCC CRL-3216) were cultured in DMEM supplemented with 10% fetal bovine serum, GlutaMAX (Thermo Fisher Scientific, USA), and penicillin–streptomycin at 37 °C and 5% CO_2_. Transfection was performed using the TurboFect reagent (Thermo Fisher Scientific, USA). For each well of a 24-well plate, 100,000 cells, 0.5 μg of plasmid DNA and 1.5 μL of TurboFect were used. Media was replaced 6 h after transfection and cells were harvested after 48 h for RNA isolation.

RNA isolation and RT-PCR: Total RNA was extracted using FreeZol Reagent (Vazyme Biotech Co., Ltd., China). Following treatment with DNase I, RNase-free (Vazyme Biotech Co., Ltd., China), cDNA synthesis was performed using the 5X RT MasMIX kit (Dialat, Moscow, Russia) with oligo(dT) primers. Splicing products were amplified using the SmarTaq polymerase (Dialat, Russia) and minigene-specific primers located in the vector exons. Amplicons were subsequently analyzed by electrophoresis in a 3% agarose gel, Sanger sequencing and fragment analysis.

### 4.6. Comparative Evaluation of Population Carrier Frequency

To evaluate the effectiveness of whole-genome sequencing in the case of autosomal recessive inherited retinal diseases, an approach based on comparing heterozygote frequencies in a cohort of patients with suspected IRDs (*n* = 1538 unrelated probands) with those in a control group of individuals without ocular phenotypes (*n* = 4151). All 1538 patients referred for genotyping to the Research Centre for Medical Genetics had previously undergone sequencing using the “Ophthalmo” gene panel. Based on these results, the proportion of patients without an established molecular diagnosis who carried single pathogenic or likely pathogenic variants in genes associated with AR-IRDs was determined. Data on carrier frequencies of pathogenic variants in the same genes in the control group were obtained from the internal laboratory records of the Research Centre for Medical Genetics; these records include whole-genome sequencing data from healthy individuals (without retinal symptoms). Comparisons of carrier frequency distributions between the two cohorts were performed using the standard Chi-square (χ^2^) test. A *p*-value < 0.05 was considered statistically significant.

### 4.7. Limitations

Segregation analysis was not performed for the majority of patients, as WGS was conducted on archival DNA and blood relatives’ samples were unavailable. This limited our ability to definitively phase compound heterozygous variants and to confirm de novo status.

No independent validation (PCR, qPCR, or Sanger sequencing) of the predicted breakpoints of the CNVs or SVs was performed. The complex chromosomal rearrangement involving *CWC27* was reconstructed from short-read sequencing data alone, without cytogenetic confirmation by karyotyping or FISH. Although the breakpoints of the identified structural variants were located within unique, non-homopolymeric regions and could therefore be mapped with nucleotide-level precision, validation of the SVs by reference methods is still required.

The depth of clinical phenotyping was limited. Given that patients were referred from different regions of Russia and the cohort initially comprised patients with isolated retinal pathology, establishing comprehensive genotype–phenotype correlations was difficult. For some patients, medical records lacked detailed data on extraocular manifestations, and information on extraocular symptoms was sometimes available only retrospectively.

Functional analysis using minigene constructs was performed only for previously undescribed variants in non-canonical splice regions. For variants located at canonical splice sites (−1/−2, +1/+2) and identified in genes in which such variants are a common cause of disease, functional analysis was not performed. In the functional study isoform identity was established by Sanger sequencing of RT-PCR products primed in the constitutive exons of the reporter vector. RNA-seq was not performed and would be complementary for quantifying residual WT versus pseudoexon-containing transcripts.

## 5. Conclusions

The present study supports the diagnostic value of WGS as an approach enabling the detection of structural rearrangements and CNVs, variants missed by targeted sequencing, and deep intronic changes that may affect splicing and require experimental validation. However, the limitations of short-read sequencing should be acknowledged, including difficulties in mapping repetitive regions and the inability to determine the *cis-/trans-* configuration without family members. In our study, a notable proportion of cases (60%) remained unresolved, indicating that further refinement of data analysis, including improvement of pipelines, or the application of other NGS approaches, such as long-read WGS, may be required. The primary value of this work lies in the identification and characterization of previously unreported variants and in the expansion of the known mutational spectrum of IRDs in the Russian population.

## Figures and Tables

**Table 1:** Spectrum of identified candidate genetic variants in the *ABCA4, USH2A, EYS, CNGB3, CRB1,* and *CEP290* genes associated with prevalent AR-IRDs in 27 patients.

Gene	№ S	P/LP Variants Previously Detected During the Panel Sequencing (hg38)	Variants Detected During WGS (hg38)	Seg.	Pub.	Class (ACMG/ AMP)
*ABCA4*	1	c.3322C>T/p.(Arg1108Cys)	NC_000001.11:g.94028925_94029978del (del 30 ex)	no	novel	LP (PVS1, PM2_Support)
	2	c.[1622T>C;3113C>T]/ p.([Leu541Pro;Ala1038Val])	c.5512del/p.(His1838Thrfs*13)	no	novel	LP (PVS1, PM2_Support)
	3	c.[1622T>C;3113C>T]/ p.([Leu541Pro;Ala1038Val])	c.4539+2064C>T	no	yes	P (rs1553189179)
	4	c.[1622T>C;3113C>T]/ p.([Leu541Pro;Ala1038Val])	c.6817-679C>A	no	yes	LP (PS3_Strong, PM2_Support, PP3_Moderate)
	5	c.4918C>T/p.(Arg1640Trp)	c.859-546G>A	no	yes	
*USH2A*	6	c.13335_13347delinsCTTG/ p.(Glu4445_Ser4449delinsAspLeu)	NC_000001.11:g.216340345_216394661del (del 4 ex)	no	yes	LP (PVS1, PM2_Support)
	7	c.13335_13347delinsCTTG/ p.(Glu4445_Ser4449delinsAspLeu)	c.7595-2144A>G	no	yes	P (rs786200928)
	8	c.13396C>T/p.(Pro4466Ser)	NC_000001.11:g.215436094_215760729del (del 57–72 ex)	no	novel	LP (PVS1, PM2_Support)
	9	c.13335_13347delinsCTTG/ p.(Glu4445_Ser4449delinsAspLeu)	NC_000001.11:g.216086061_216149817del (del 22–24 ex)	no	yes	P (PVS1, PM2_Support, PM3_Strong
	10	c.8682-9A>G	NC_000001.11:g.216086061_216149817del (del 22–24 ex)	no	yes	
	11	c.11864G>A/p.(Trp3955*)	NC_000001.11:g.216086061_216149817del (del 22–24 ex)	comp	yes	
	12	c.11864G>A/p.(Trp3955*)	NC_000001.11:g.216086061_216149817del (del 22–24 ex)	no	yes	
	13	c.4174G>T/p.(Gly1392*)	c.5573-11C>G	comp	novel	LP (PS3_Support, PM2_Support, PM3_Strong, PP3_Support)
*EYS*	14	c.8133_8137del/p.(Phe2712Cysfs*33)	NC_000006.12:g.65701926_66236163delins65834315_66235495inv	no	novel	LP (PVS1, PM2_Support)
	15	c.8648_8655del/p.(Thr2883Lysfs*4)	NC_000006.12:g.64877752_64886823del (del 19 ex)	no	novel	LP (PVS1, PM2_Support)
	16	c.4815_4816delinsATAG/p.(Thr1606*)	NC_000006.12:g.64877752_64886823del (del 19 ex)	no	novel	
	17	c.7276T>G/p.(Tyr2426Asp)	NC_000006.12:g.65455041_65636071delins 65634428_65635747inv	no	novel	LP (PVS1, PM2_Support)
	18	c.[1155T>A;8648_8655del]/p.([Cys385*;Thr2883Lysfs*4)	NC_000006.12:g.64877752_64886823del (del 19 ex)	no	novel	LP (PVS1, PM2_Support)
	19	c.8133_8137del/p.(Phe2712Cysfs*33)	NC_000006.12:g.64877752_64886823del (del 19 ex)	no	novel	
	20	c.2058_2061dup/p.(His688Phefs*12)	NC_000006.12:g.64731274_64815790dup (dup 22 ex)	no	novel	LP (PVS1, PM2_Support)
	21	c.5927+1G>T	NC_000006.12:g.65024915_65781302delins 65024941_65781255inv	no	novel	LP (PVS1, PM2_Support)
	22	c.8648_8655del/p.(Thr2883Lysfs*4*)	c.2381+824C>A	no	novel	LP (PS3_Moderate, PM2_Support, PP3_Support)
*CNGB3*	23	c.819_826del/p.(Arg274Valfs*13)	NC_000008.11:g.86492499_86588520delins 86577137_86588520inv	comp	yes	LP (PVS1_Strong, PM3_Strong, PM2_Support)
	24	c.819_826del/p.(Arg274Valfs*13)	NC_000008.11:g.86598578_86606871del (del 15 ex)	no	yes	P (PVS1, PM2_Support, PM3_Strong)
	25	c.1006G>T/p.(Glu336*)	NC_000008.11:g.86598578_86606871del (del 15 ex)	no	yes	
*CRB1*	26	c.2536G>A/p.(Gly846Arg)	NC_000001.11:g.197429490_197431630el (del 8 ex)	no	novel	LP (PVS1, PM2_Support)
*CEP290*	27	c.1334_1339del/ p.(Leu445_Lys446del)	c.2818-955A>G	no	novel	LP (PS3_Strong, PM2_Support, PP3_Support)

Note: reference transcripts: *ABCA4* (NM_000350.3), *USH2A* (NM_206933.3), *EYS* (NM_001142800.2), *CNGB3* (NM_019098.4), *CRB1* (NM_201253.3), *CEP290* (NM_025114.4). Abbreviations: P—pathogenic, LP—likely pathogenic, S—subject, Seg.—Sanger segregation analysis, Pub.—published.

**Table 2:** Spectrum of detected candidate genetic variants associated with various retinal disorders in 16 patients.

Gene	№S	Variants Detected During WGS (hg38)	Zyg.	Seg.	Pub.	Class (ACMG/AMP)	Phenotype (OMIM)
	Autosomal recessive retinal diseases						
*BBS5*	28	c.80del/p.(Gly27Glufs*8)	hom	no	novel	LP (PVS1, PM2_Support)	Bardet–Biedl syndrome 5 (OMIM:615983)
*BBS10*	29	c.145C>T/p.(Arg49Trp) c.271dup/p.(Cys91Leufs*5)	het het	no	yes yes	P (rs768933093, rs549625604)	Bardet–Biedl syndrome 10 (OMIM:615987)
*ALMS1*	30	c.5690C>G/p.(Ser1897*)	hom	no	novel	LP (PVS1, PM2_Support)	Alstrom syndrome (OMIM:203800)
*TRPM1*	31	c.965+29G>A chr15:31052262_31084941dup (dup 2–11 ex)	het het	no	yes novel	LP (PS3_Strong, PM2_Support, PP3_Support) LP (PVS1, PM2_Support)	Night blindness, congenital stationary 1C (OMIM:613216)
*PROM1*	32	c.1301+2T>C chr4:16015841_16023179del (del 9–10 ex)	het het	no	yes novel	P (rs775957498) LP (PVS1, PM2_Support)	Retinitis pigmentosa 41 (OMIM:612095)
*TTLL5*	33	c.524dup/p.(Pro176Thrfs*37) c.1513A>G/p.(Met505Val)	het het	no	novel yes	LP (PVS1, PM2_Support) LP (PM2_Support, PM3_Strong, PP3_Support) (rs781316021)	Cone-rod dystrophy 19 (OMIM:615860)
*CWC27*	34	c.1002dup/p.(Val335Serfs*13) Complex rearrangement: NC_000005.10:g.24496826_qterdelins[(?_27231145)_(64818765_?)inv; NC_000014.9:g.(?_73369171_qter)]; NC_000014.9:g.73369171_qterdelins [NC_000008.11:g.pter_(31329176_?)inv]; NC_000008.11:g.pter_(31329176_?)delins[NC_000005.10:g.(24496827_27228425;(?_64818766)_qter)inv]	het het	no	yes novel	P (rs752159903) LP *	Retinitis pigmentosa with or without skeletal anomalies (OMIM:250410)
	Autosomal dominant retinal diseases						
*PRPF31*	35	chr19:54111608_54116988del (del 1 ex)	het	no	yes	LP (PVS1, PM2_Support)	Retinitis pigmentosa 11 (OMIM:600138)
*COL11A1*	36	c.2502+1del	het	no	novel	LP (PVS1, PM2_Support)	Stickler/Marshall syndrome (OMIM:604841, OMIM:154780)
	37	c.4972-42_4972delinsTTTATA	het	no	novel	LP (PVS1, PM2_Support)	
	X-linked retinal diseases						
*RPGR*	38	c.1926dup/p.(Ser643Ilefs*10)	het	no	yes	LP (PVS1, PM2_Support)	Cone-rod dystrophy, 1 (OMIM:304020), RP3, (OMIM:300029)
*NYX*	39	c.903_904del/p.(Leu302Alafs*185)	hemi	no	novel	LP (PVS1, PM2_Support)	Night blindness, congenital stationary 1A, (OMIM:310500)
*RP2*	40	NC_000023.11:g.46551711_46839408 delins46679500_46766119inv	hemi	no	novel	LP (PVS1, PM2_Support)	Retinitis pigmentosa 2 (OMIM:312600)
*CHM*	41	c.1245-521A>G	hemi	no	novel	LP (PS3_Strong, PM2_Support, PP3_Support)	Choroideremia (OMIM:303100)
	Mitochondrial diseases						
*MT-ATP6*	42	NC_012920.1:m.8993T>G (heteroplasmy 84%)		no	yes	repeatedly described as P (rs199476133, rs199476104)	MILS (OMIM:500017), NARP (OMIM:551500)
*MT-ND6*	43	NC_012920.1:m.14484T>C (homoplasmy) **		no	yes		LHON (OMIM:535000)

Note: reference transcripts: *BBS5* (NM_152384.3), *BBS10* (NM_024685.4), *ALMS1* (NM_015120.4), *TRPM1* (NM_001252024.2), *PROM1* (NM_006017.3), *TTLL5* (NM_015072.5), *CWC27* (NM_005869.4), *PRPF31* (NM_015629.4), *COL11A1* (NM_001854.4), *RPGR* (NM_001034853.2), *NYX* (NM_001378477.3), *RP2* (NM_006915.3), *CHM* (NM_000390.4). Abbreviations: Zyg.—zygosity (hom—homozygous, het—heterozygous, hemi—hemizygous); P—pathogenic; LP—likely pathogenic, S—subject, Seg.—Sanger segregation analysis. * This variant was interpreted as likely pathogenic based on molecular and clinical data; karyotyping was not performed. ** The m.14484T>C variant in the homoplasmic state is associated with classic Leber’s hereditary optic neuropathy (LHON); however, its penetrance may vary depending on genetic factors determining the mitochondrial haplogroup as well as environmental factor.

## Data Availability

The original contributions presented in this study are included in the article/Supplementary Materials. Further inquiries can be directed to the corresponding author.

## Supplementary Materials

The following supporting information can be downloaded at: https://www.mdpi.com/article/10.3390/ijms27209022/s1.

## Abbreviations

The following abbreviations are used in this manuscript:

AR	autosomal recessive
CNV	copy number variant
DIV	deep intronic variant
IRDs	inherited retinal diseases
LINE	long interspersed nuclear element
NHEJ	non-homologous end joining
NMD	nonsense-mediated decay
SINE	short interspersed nuclear element
VUS	variant of uncertain significance
WES	whole-exome sequencing
WGS	whole-genome sequencing

## References

[1] Hanany M., Shalom S., Ben-Yosef T., Sharon D. (2024). Comparison of Worldwide Disease Prevalence and Genetic Prevalence of Inherited Retinal Diseases and Variant Interpretation Considerations. Cold Spring Harb. Perspect. Med..

[2] Gocuk S.A., Jiao Y., Britten-Jones A.C., Kerr N.M., Lim L., Skalicky S., Stawell R., Ayton L.N., Mack H.G. (2022). Genetic Testing of Inherited Retinal Disease in Australian Private Tertiary *Ophthalmol.* Practice. Clin. Ophthalmol..

[3] Shah M., Shanks M., Packham E., Williams J., Haysmoore J., MacLaren R.E., Németh A.H., Clouston P., Downes S.M. (2020). Next Generation Sequencing Using Phenotype-Based Panels for Genetic Testing in Inherited Retinal Diseases. Ophthalmic Genet..

[4] Rattner A., Smallwood P.M., Williams J., Cooke C., Savchenko A., Lyubarsky A., Pugh E.N., Nathans J. (2001). A Photoreceptor-Specific Cadherin Is Essential for the Structural Integrity of the Outer Segment and for Photoreceptor Survival. Neuron.

[5] Weisschuh N., Obermaier C.D., Battke F., Bernd A., Kuehlewein L., Nasser F., Zobor D., Zrenner E., Weber E., Wissinger B. (2020). Genetic Architecture of Inherited Retinal Degeneration in Germany: A Large Cohort Study from a Single Diagnostic Center over a 9-Year Period. Hum. Mutat..

[6] Ordonez-Labastida V., Montes-Almanza L., Garcia-Martinez F., Zenteno J.C. (2022). Effectiveness of Whole-Exome Sequencing for the Identification of Causal Mutations in Patients with Suspected Inherited Ocular Diseases. Rev. Investig. Clínica.

[7] Yavas C., Arvas Y.E., Dogan M., Gezdirici A., Aslan E.S., Karapapak M., Barıs S., Eroz R. (2025). Revealing Molecular Diagnosis With Whole Exome Sequencing in Patients With Inherited Retinal Disorders. Clin. Genet..

[8] Carss K.J., Arno G., Erwood M., Stephens J., Sanchis-Juan A., Hull S., Megy K., Grozeva D., Dewhurst E., Malka S. (2017). Comprehensive Rare Variant Analysis via Whole-Genome Sequencing to Determine the Molecular Pathology of Inherited Retinal Disease. Am. J. Hum. Genet..

[9] Bryant L., Lozynska O., Maguire A., Aleman T., Bennett J. (2017). Prescreening Whole Exome Sequencing Results from Patients with Retinal Degeneration for Variants in Genes Associated with Retinal Degeneration. OPTH.

[10] Ma D.J., Lee H.-S., Kim K., Choi S., Jang I., Cho S.-H., Yoon C.K., Lee E.K., Yu H.G. (2021). Whole-Exome Sequencing in 168 Korean Patients with Inherited Retinal Degeneration. BMC Med. Genom..

[11] Hussain H.M.J., Wang M., Huang A., Schmidt R., Qian X., Yang P., Marra M., Li Y., Pennesi M.E., Chen R. (2023). Novel Pathogenic Mutations Identified from Whole-Genome Sequencing in Unsolved Cases of Patients Affected with Inherited Retinal Diseases. Genes.

[12] Maggi J., Koller S., Feil S., Bachmann-Gagescu R., Gerth-Kahlert C., Berger W. (2024). Limited Added Diagnostic Value of Whole Genome Sequencing in Genetic Testing of Inherited Retinal Diseases in a Swiss Patient Cohort. IJMS.

[13] Liu X., Hu F., Zhang D., Li Z., He J., Zhang S., Wang Z., Zhao Y., Wu J., Liu C. (2024). Whole Genome Sequencing Enables New Genetic Diagnosis for Inherited Retinal Diseases by Identifying Pathogenic Variants. npj Genom. Med..

[14] Ogorodova N., Stepanova A., Kadyshev V., Kuznetsova S., Ismagilova O., Chukhrova A., Polyakov A., Kutsev S., Shchagina O. (2024). A Comparative Evaluation of the Genetic Variant Spectrum in the USH2A Gene in Russian Patients with Isolated and Syndromic Forms of Retinitis Pigmentosa. IJMS.

[15] Ogorodova N.Y., Stepanova A.A., Shchagina O.A. (2024). Frequent Genetic Variants of Autosomal Recessive Nonsyndromic Forms of Inherited Retinal Diseases in the Russian Federation Russ. J. Genet..

[16] Pieras J.I., Barragán I., Borrego S., Audo I., González-Del Pozo M., Bernal S., Baiget M., Zeitz C., Bhattacharya S.S., Antiñolo G. (2011). Copy-Number Variations in EYS: A Significant Event in the Appearance of arRP. Investig. Ophthalmol. Vis. Sci..

[17] Gao F.-J., Li J.-K., Chen H., Hu F.-Y., Zhang S.-H., Qi Y.-H., Xu P., Wang D.-D., Wang L.-S., Chang Q. (2019). Genetic and Clinical Findings in a Large Cohort of Chinese Patients with Suspected Retinitis Pigmentosa. Ophthalmology.

[18] Quinodoz M., Kaminska K., Cancellieri F., Han J.H., Peter V.G., Celik E., Janeschitz-Kriegl L., Schärer N., Hauenstein D., György B. (2024). Detection of Elusive DNA Copy-Number Variations in Hereditary Disease and Cancer through the Use of Noncoding and off-Target Sequencing Reads. Am. J. Hum. Genet..

[19] Eisenberger T., Neuhaus C., Khan A.O., Decker C., Preising M.N., Friedburg C., Bieg A., Gliem M., Charbel Issa P., Holz F.G. (2013). Increasing the Yield in Targeted Next-Generation Sequencing by Implicating CNV Analysis, Non-Coding Exons and the Overall Variant Load: The Example of Retinal Dystrophies. PLoS ONE.

[20] Shatokhina O., Galeeva N., Stepanova A., Markova T., Lalayants M., Alekseeva N., Tavarkiladze G., Markova T., Bessonova L., Petukhova M. (2022). Spectrum of Genes for Non-GJB2-Related Non-Syndromic Hearing Loss in the Russian Population Revealed by a Targeted Deafness Gene Panel. Int. J. Mol. Sci..

[21] de Bruijn S.E., Rodenburg K., Corominas J., Ben-Yosef T., Reurink J., Kremer H., Whelan L., Plomp A.S., Berger W., Farrar G.J. (2023). Optical Genome Mapping and Revisiting Short-Read Genome Sequencing Data Reveal Previously Overlooked Structural Variants Disrupting Retinal Disease-Associated Genes. Genet. Med..

[22] Neuhaus C., Eisenberger T., Decker C., Nagl S., Blank C., Pfister M., Kennerknecht I., Müller-Hofstede C., Charbel Issa P., Heller R. (2017). Next-Generation Sequencing Reveals the Mutational Landscape of Clinically Diagnosed Usher Syndrome: Copy Number Variations, Phenocopies, a Predominant Target for Translational Read-through, and PEX26 Mutated in Heimler Syndrome. Mol. Genet. Genom. Med..

[23] Alfares A., Alfadhel M., Wani T., Alsahli S., Alluhaydan I., Al Mutairi F., Alothaim A., Albalwi M., Al Subaie L., Alturki S. (2017). A Multicenter Clinical Exome Study in Unselected Cohorts from a Consanguineous Population of Saudi Arabia Demonstrated a High Diagnostic Yield. Mol. Genet. Metab..

[24] Faqeih E.A., Almannai M., Saleh M.M., AlWadei A.H., Samman M.M., Alkuraya F.S. (2018). Phenotypic Characterization of KCTD3-Related Developmental Epileptic Encephalopathy. Clin. Genet..

[25] Bonnet C., Riahi Z., Chantot-Bastaraud S., Smagghe L., Letexier M., Marcaillou C., Lefèvre G.M., Hardelin J.-P., El-Amraoui A., Singh-Estivalet A. (2016). An Innovative Strategy for the Molecular Diagnosis of Usher Syndrome Identifies Causal Biallelic Mutations in 93% of European Patients. Eur. J. Hum. Genet..

[26] Lin S., Vermeirsch S., Pontikos N., Martin-Gutierrez M.P., Daich Varela M., Malka S., Schiff E., Knight H., Wright G., Jurkute N. (2024). Spectrum of Genetic Variants in the Most Common Genes Causing Inherited Retinal Disease in a Large Molecularly Characterized United Kingdom Cohort. Ophthalmol. Retin..

[27] Mayer A.K., Van Cauwenbergh C., Rother C., Baumann B., Reuter P., De Baere E., Wissinger B., Kohl S., ACHM Study Group (2017). CNGB3 Mutation Spectrum Including Copy Number Variations in 552 Achromatopsia Patients. Hum. Mutat..

[28] Bauwens M., Garanto A., Sangermano R., Naessens S., Weisschuh N., De Zaeytijd J., Khan M., Sadler F., Balikova I., Van Cauwenbergh C. (2019). ABCA4-Associated Disease as a Model for Missing Heritability in Autosomal Recessive Disorders: Novel Noncoding Splice, Cis-Regulatory, Structural, and Recurrent Hypomorphic Variants. Genet. Med..

[29] Bertrand R.E., Wang J., Li Y., Cheng X., Wang K., Stoilov P., Chen R. (2022). Cwc27, Associated with Retinal Degeneration, Functions as a Splicing Factor in Vivo. Hum. Mol. Genet..

[30] Zhang L., Wang T., Wright A.F., Suri M., Schwartz C.E., Stevenson R.E., Valle D. (2006). A Microdeletion in Xp11.3 Accounts for Co-Segregation of Retinitis Pigmentosa and Mental Retardation in a Large Kindred. Am. J. Med. Genet. A.

[31] Nassisi M., Mohand-Saïd S., Andrieu C., Antonio A., Condroyer C., Méjécase C., Varin J., Wohlschlegel J., Dhaenens C.-M., Sahel J.-A. (2019). Prevalence of ABCA4 Deep-Intronic Variants and Related Phenotype in An Unsolved “One-Hit” Cohort with Stargardt Disease. Int. J. Mol. Sci..

[32] Reurink J., Weisschuh N., Garanto A., Dockery A., van den Born L.I., Fajardy I., Haer-Wigman L., Kohl S., Wissinger B., Farrar G.J. (2023). Whole Genome Sequencing for USH2A-Associated Disease Reveals Several Pathogenic Deep-Intronic Variants That Are Amenable to Splice Correction. HGG Adv..

[33] Vaché C., Besnard T., le Berre P., García-García G., Baux D., Larrieu L., Abadie C., Blanchet C., Bolz H.J., Millan J. (2012). Usher Syndrome Type 2 Caused by Activation of an USH2A Pseudoexon: Implications for Diagnosis and Therapy. Hum. Mutat..

[34] Dreyer B., Tranebjaerg L., Rosenberg T., Weston M.D., Kimberling W.J., Nilssen O. (2000). Identification of Novel USH2A Mutations: Implications for the Structure of USH2A Protein. Eur. J. Hum. Genet..

[35] Zhou L., Xiao X., Li S., Jia X., Wang P., Sun W., Zhang F., Li J., Li T., Zhang Q. (2018). Phenotypic Characterization of Patients with Early-Onset High Myopia Due to Mutations in COL2A1 or COL11A1: Why Not Stickler Syndrome?. Mol. Vis..

[36] Zobor D., Brühwiler B., Zrenner E., Weisschuh N., Kohl S. (2023). Genetic and Clinical Profile of Retinopathies Due to Disease-Causing Variants in Leber Congenital Amaurosis (LCA)-Associated Genes in a Large German Cohort. IJMS.

[37] Andersen M.K.G., Bertelsen M., Grønskov K., Kohl S., Kessel L. (2023). Genetic and Clinical Characterization of Danish Achromatopsia Patients. Genes.

[38] Koyanagi Y., Akiyama M., Nishiguchi K.M., Momozawa Y., Kamatani Y., Takata S., Inai C., Iwasaki Y., Kumano M., Murakami Y. (2019). Genetic Characteristics of Retinitis Pigmentosa in 1204 Japanese Patients. J. Med. Genet..

[39] Xu K., Xie Y., Sun T., Zhang X., Chen C., Li Y. (2020). Genetic and Clinical Findings in a Chinese Cohort with Leber Congenital Amaurosis and Early Onset Severe Retinal Dystrophy. Br. J. Ophthalmol..

[40] Stepanova A., Ogorodova N., Kadyshev V., Shchagina O., Kutsev S., Polyakov A. (2023). A Molecular Genetic Analysis of RPE65-Associated Forms of Inherited Retinal Degenerations in the Russian Federation. Genes.

[41] Perez-Carro R., Corton M., Sánchez-Navarro I., Zurita O., Sanchez-Bolivar N., Sánchez-Alcudia R., Lelieveld S.H., Aller E., Lopez-Martinez M.A., López-Molina M.I. (2016). Corrigendum: Panel-Based NGS Reveals Novel Pathogenic Mutations in Autosomal Recessive Retinitis Pigmentosa. Sci. Rep..

[42] Van Schil K., Naessens S., Van de Sompele S., Carron M., Aslanidis A., Van Cauwenbergh C., Kathrin Mayer A., Van Heetvelde M., Bauwens M., Verdin H. (2018). Mapping the Genomic Landscape of Inherited Retinal Disease Genes Prioritizes Genes Prone to Coding and Noncoding Copy-Number Variations. Genet. Med..

[43] Kamenarova K., Mihova K., Veleva N., Mermeklieva E., Mihaylova B., Dimitrova G., Oscar A., Shandurkov I., Cherninkova S., Kaneva R. (2022). Panel-based Next-generation Sequencing Identifies Novel Mutations in Bulgarian Patients with Inherited Retinal Dystrophies. Molec Gen. Gen. Med..

[44] Maltese P.E., Colombo L., Martella S., Rossetti L., El Shamieh S., Sinibaldi L., Passarelli C., Coppè A.M., Buzzonetti L., Falsini B. (2022). Genetics of Inherited Retinal Diseases in Understudied Ethnic Groups in Italian Hospitals. Front. Genet..

[45] Dad S., Rendtorff N.D., Kann E., Albrechtsen A., Mehrjouy M.M., Bak M., Tommerup N., Tranebjærg L., Rosenberg T., Jensen H. (2015). Partial USH2A Deletions Contribute to Usher Syndrome in Denmark. Eur. J. Hum. Genet..

[46] McClinton B., Crinnion L.A., McKibbin M., Mukherjee R., Poulter J.A., Smith C.E.L., Ali M., Watson C.M., Inglehearn C.F., Toomes C. (2023). Targeted Nanopore Sequencing Enables Complete Characterisation of Structural Deletions Initially Identified Using Exon-Based Short-Read Sequencing Strategies. Mol. Genet. Genom. Med..

[47] Slijkerman R.W., Vaché C., Dona M., García-García G., Claustres M., Hetterschijt L., Peters T.A., Hartel B.P., Pennings R.J., Millan J.M. (2016). Antisense Oligonucleotide-Based Splice Correction for USH2A-Associated Retinal Degeneration Caused by a Frequent Deep-Intronic Mutation. Mol. Ther. Nucleic Acids.

[48] Cheng S., Xing C., Chen Z., Zhang S., Chen W. (2025). Clinical Trial Landscape of Gene Therapy for Retinal Degenerative Diseases: An Analysis Based on the Trialtrove Database. Stem Cell Res. Ther..

[49] Estrada-Cuzcano A., Koenekoop R.K., Senechal A., De Baere E.B.W., de Ravel T., Banfi S., Kohl S., Ayuso C., Sharon D., Hoyng C.B. (2012). BBS1 Mutations in a Wide Spectrum of Phenotypes Ranging from Nonsyndromic Retinitis Pigmentosa to Bardet-Biedl Syndrome. Arch. Ophthalmol..

[50] Manichaikul A., Mychaleckyj J.C., Rich S.S., Daly K., Sale M., Chen W.M. (2010). Robust relationship inference in genome-wide association studies. Bioinformatics.

[51] Richards S., Aziz N., Bale S., Bick D., Das S., Gastier-Foster J., Grody W.W., Hegde M., Lyon E., Spector E. (2015). Standards and Guidelines for the Interpretation of Sequence Variants: A Joint Consensus Recommendation of the American College of Medical Genetics and Genomics and the Association for Molecular Pathology. Genet. Med..

[52] Riggs E.R., Andersen E.F., Cherry A.M., Kantarci S., Kearney H., Patel A., Raca G., Ritter D.I., South S.T., Thorland E.C. (2020). Technical standards for the interpretation and reporting of constitutional copy-number variants: A joint consensus recommendation of the American College of Medical Genetics and Genomics (ACMG) and the Clinical Genome Resource (ClinGen). Genet. Med..

[53] Chen X., Schulz-Trieglaff O., Shaw R., Barnes B., Schlesinger F., Källberg M., Cox A.J., Kruglyak S., Saunders C.T. (2016). Manta: Rapid Detection of Structural Variants and Indels for Germline and Cancer Sequencing Applications. Bioinformatics.

[54] Yeo G., Burge C.B. (2004). Maximum Entropy Modeling of Short Sequence Motifs with Applications to RNA Splicing Signals. J. Comput. Biol..

[55] Bychkov I., Kuznetsova A., Baydakova G., Gorobets L., Kenis V., Dimitrieva A., Filatova A., Tabakov V., Skoblov M., Zakharova E. (2022). Processed pseudogene insertion in GLB1 causes Morquio B disease by altering intronic splicing regulatory landscape. npj Genom. Med..

