# Supplementary material for: Whole-Genome Sequencing in Diagnostics of Unsolved Cases of Inherited Retinal Diseases
Source: Int J Mol Sci. 2026 Oct 12;27(20):9022. doi: 10.3390/ijms27209022 (PMC13704990; doi:10.3390/ijms27209022)
Supplement: Supplementary file 1 [file ijms-27-09022-s001.zip › Suppl S1.pdf]

Supplementary S1. IRD-associated genes screened using custom panel «Ophthalmo»

ABCA4, ADAMTSL4, COL8A2, CRB1, EPHA2, FOXE3, GJA8, GNAT2, HMCN1, MYOC, NMNAT1, PLA2G5, PRPF3, RD3, RPE65, SEMA4A, UBIAD1, USH2A, ZNF644, ARMS2, CDHR1, HTRA1, OAT, OPTN, PAX2, PDE6C, PITX3, RAB18, RBP3, RBP4, RGR, SLC16A12, VIM, ZEB1, BEST1, CABP4, CAPN5, CEP164, CRYAB, FZD4, LRP5, MFRP, PAX6, ROM1, TEAD1, TMEM126A, ZNF408, CEP290, DCN, GDF3, KERA, KIF21A, KRT3, MIP, PDE6H, RDH5, GJA3, GRK1, RB1, LTBP2, NRL, OTX2, RDH12, SIX6, SMOC1, SPATA7, TTC8, VSX2, ALDH1A3, NR2E3, OCA2, POLG, SLC24A1, STRA6, TRPM1, ABCC6, ARL2BP, BBS2, CHST6, CLN3, CNGB1, HSF4, MAF, SLC38A8, TUBB3, AIPL1, CA4, CRYBA1, FSCN2, GPR179, GUCY2D, KRT12, PDE6G, PITPNM3, POLG2, PRCD, PRPF8, RGS9, UNC45B, RAX, TCF4, CRX, LIM2, NTF4, OPA3, PRPF31, RGS9BP, SIPA1L3, ABCB6, C2orf71, CERKL, CHN1, CNGA3, CNNM4, CRYBA2, CRYGC, CRYGD, CRYGB, CYP1B1, EFEMP1, FAM161A, IFT172, KCNJ13, MERTK, PIKFYVE, PRSS56, RAB3GAP1, SAG, SNRNP200, TTC21B, ZNF513, CHMP4B, IDH3B, PRPF6, SLC4A11, VSX1, CRYAA, LSS, CRYBA4, CRYBB1, CRYBB2, CRYBB3, TIMP3, ARL6, BFSP2, CLRN1, CRYGS, FYCO1, GNAT1, IMPG2, OPA1, RHO, SLC7A14, SOX2, CNGA1, CYP4V2, LRAT, LRIT3, PDE6B, PITX2, PROM1, RAB28, SLC25A4, TENM3, WFS1, GRM6, PDE6A, WDR36, COL11A2, ELOVL4, EYS, FOXC1, GCNT2, GUCA1A, IMPG1, LCA5, MAK, PRPH2, RIMS1, TULP1, AGK, IMPDH1, KLHL7, OPN1SW, RP9, SHH, TSPAN12, ADAM9, C8orf37, CNGB3, GDF6, HGSNAT, RP1, RP1L1, KCNV2, PRPF4, TDRD7, TOPORS, CACNA1F, CHM, CHRDL1, FRMD7, GPR143, NDP, NHS, OFD1, OPN1LW, OPN1MW, RP2, RPGR, RLBP1, RS1
