# Supplementary material for: Whole-Genome Sequencing in Diagnostics of Unsolved Cases of Inherited Retinal Diseases
Source: Int J Mol Sci. 2026 Oct 12;27(20):9022. doi: 10.3390/ijms27209022 (PMC13704990; doi:10.3390/ijms27209022)
Supplement: Supplementary file 1 [file ijms-27-09022-s001.zip › Suppl S2.pdf]

Supplementary S2. Oligonucleotide primers used in the functional assay

Primers used for minigene constructions

Target	Forward primer	Reverse primer
USH2A	5’-tcttctgtaggctgatgtaactc	5’-agagaataccttgatggaaacacgtc
EYS	5’-ggctggcattatctaactcaactct	5’- gaaataaagcaacatactgacagcca
CEP290	5’-gacttacctacatacttcatgcccata	5’-tgggagaatagtttgttctgggtac
CHM	10 exon 5’-gtccatttcaaggatgttgccctt	10 exon 5’-taccttcgcttgctaatgggtgga
	11 exon 5’-tccacccattagcaagcgaaggta	11 exon 5’-aatggggagacgttggtcaaagg

Note: the CHM insert was amplified as two overlapping fragments to include additional flanking context; the other inserts were amplified as a single fragment.

Primers used for site-directed mutagenesis

Target	Forward primer	Reverse primer
USH2A:<br>*c.5573-11C>G*	5’-caaaatgtagtgctgcagaggac	5’-ggaaggactattaccctgaagc
EYS:<br>*c.2381+824C>A*	5’-ttctgagtaggtgtttatgtattatgcc	5’- gttgtagttgcttttgtggactc
CEP290:<br>*c.2818-955A>G*	5’-ggttgtaatgggtgatgtcaggc	5’-ggagaagaaaggctgccaaaatg
CHM:<br>*c.1245-521A>G*	5’-cagagacctgataatgtccgtgc	5’-ggcctttacaggctctgaatgg

Primers used for RT-PCR and Sanger sequencing of minigene cDNA

TFP_F<br>(constitutive 5′ exon of the reporter)	5’-acaaagagacctacgtcgagca
GFP_R<br>(constitutive 3′ exon of the reporter)	5’-agctcgatcagcacgggcacgat
